# Core and Differentially Abundant Bacterial Taxa in the Rhizosphere of Field Grown *Brassica napus* Genotypes: Implications for Canola Breeding

**DOI:** 10.3389/fmicb.2019.03007

**Published:** 2020-01-15

**Authors:** Zelalem M. Taye, Bobbi L. Helgason, Jennifer K. Bell, Charlotte E. Norris, Sally Vail, Stephen J. Robinson, Isobel A. P. Parkin, Melissa Arcand, Steven Mamet, Matthew G. Links, Tanner Dowhy, Steven Siciliano, Eric G. Lamb

**Affiliations:** ^1^Department of Plant Sciences, College of Agriculture and Bioresources, University of Saskatchewan, Saskatoon, SK, Canada; ^2^Department of Soil Science, College of Agriculture and Bioresources, University of Saskatchewan, Saskatoon, SK, Canada; ^3^Saskatoon Research and Development Centre, Agriculture and Agri-Food Canada, Saskatoon, SK, Canada; ^4^Department of Computer Science, College of Arts and Science, University of Saskatchewan, Saskatoon, SK, Canada; ^5^Department of Animal and Poultry Science, College of Agriculture and Bioresources, University of Saskatchewan, Saskatoon, SK, Canada

**Keywords:** *Brassica napus*, breeding, canola, core microbiome, differential abundance, microbiome, plant–microbial interactions, rhizosphere

## Abstract

Modifying the rhizosphere microbiome through targeted plant breeding is key to harnessing positive plant–microbial interrelationships in cropping agroecosystems. Here, we examine the composition of rhizosphere bacterial communities of diverse *Brassica napus* genotypes to identify: (1) taxa that preferentially associate with genotypes, (2) core bacterial microbiota associated with *B. napus*, (3) heritable alpha diversity measures at flowering and whole growing season, and (4) correlation between microbial and plant genetic distance among canola genotypes at different growth stages. Our aim is to identify and describe signature microbiota with potential positive benefits that could be integrated in *B. napus* breeding and management strategies. Rhizosphere soils of 16 diverse genotypes sampled weekly over a 10-week period at single location as well as at three time points at two additional locations were analyzed using 16S rRNA gene amplicon sequencing. The *B. napus* rhizosphere microbiome was characterized by diverse bacterial communities with 32 named bacterial phyla. The most abundant phyla were Proteobacteria, Actinobacteria, and Acidobacteria. Overall microbial and plant genetic distances were highly correlated (*R* = 0.65). Alpha diversity heritability estimates were between 0.16 and 0.41 when evaluated across growth stage and between 0.24 and 0.59 at flowering. Compared with a reference *B. napus* genotype, a total of 81 genera were significantly more abundant and 71 were significantly less abundant in at least one *B. napus* genotype out of the total 558 bacterial genera. Most differentially abundant genera were Proteobacteria and Actinobacteria followed by Bacteroidetes and Firmicutes. Here, we also show that *B. napus* genotypes select an overall core bacterial microbiome with growth-stage-related patterns as to how taxa joined the core membership. In addition, we report that sets of *B. napus* core taxa were consistent across our three sites and 2 years. Both differential abundance and core analysis implicate numerous bacteria that have been reported to have beneficial effects on plant growth including disease suppression, antifungal properties, and plant growth promotion. Using a multi-site year, temporally intensive field sampling approach, we showed that small plant genetic differences cause predictable changes in canola microbiome and are potential target for direct and indirect selection within breeding programs.

## Introduction

The plant microbiome is a promising avenue of exploration to enhance crop productivity and management. Recent studies are revealing that plant breeding can shape the composition of root-associated bacterial communities including enhancing antagonistic potential toward pathogens (Peiffer and Ley, 2013; Bouffaud et al., 2014; Cardinale et al., 2015). Promising results that indicate both microbiome heritability and associations with yield have been reported. For example, a study on 27 maize inbred lines indicated the presence of a small but significant proportion of heritable variation in total bacterial diversity across field environments and substantially more heritable variation between replicates of lines within each field (Peiffer et al., 2013). A study of *Brassica napus* root-associated microbiomes in the Canadian Prairies identified bacterial taxa which were positively correlated with canola yield (Lay et al., 2018). Identifying genetically controlled positive plant–microbial interactions by comparing lines within breeding programs and across diversity panels is the first step in determining if plant breeders could develop varieties by selecting for genetic factors controlling beneficial plant–microbial interrelationships.

The rhizosphere microbiome has commonly been targeted to identify positive plant–microbial relationships. Past microbiome studies using culture dependent and independent approaches have shed light on the structure and composition of the plant microbiota in many crops (Garland, 1996; Germida et al., 1998; Bulgarelli et al., 2012; Lundberg et al., 2012; Peiffer et al., 2013; Schlaeppi et al., 2013; Edwards et al., 2015; Lay et al., 2018). Continuing advances in biotechnology and bioinformatics are enabling researchers to evaluate the microbiome to a greater depth, incorporating more replications and to account for variables such as genotype, time, and space.

Our goal was to characterize the core rhizosphere microbiome and identify contrasting components of the bacterial microbiota between plant genotypes of *B. napus* (canola) grown under field conditions. Our specific objectives were to: (1) identify bacterial taxa differentially abundant between multiple canola genotypes, (2) screen taxa that were differentially abundant between genotypes to identify potential beneficial plant–microbial interactions, (3) characterize the core canola rhizosphere bacterial microbiota across the full growing season at a single site, (4) examine the consistency of the core microbiota across years and location, (5) estimate the heritability of alpha diversity measures at flowering and for the whole growing season, and (6) determine correlation between the microbial and plant genetic distances among canola genotypes at different growth stages.

## Materials and Methods

### Site Description

Experiments were conducted in 2016 at a single location and at three locations in 2017. The 2016 experimental site was near Saskatoon, SK, Canada (latitude 52.181366, longitude -106.502941). The soil is a haplic Kastanozem (Iuss Working Group WRB, 2015), with a clay loam texture. The field was managed with an oilseed, wheat, barley, fallow rotation with fallow in the growing season prior to this experiment. Pre-trial field nutrient assessments guided pre-trial fertilizer applications of 78.5 kg ha^–1^ NH_3_ in the fall of 2015 with N21.5, P35 S25 being applied prior to planting in the spring of 2016. EdgeTM (Ethalfluralin – Group 3 – dinitroanaline) (Gowan Company, Yuma, AZ, United States) a pre-emergence herbicide, was also applied to the field at a rate of 19.1 kg ha^–1^ for weed control. The 2017 locations were at Saskatoon (latitude 52.183149, longitude -106.514904), Melfort (latitude 52.819333, longitude −104.596348), and Scott (latitude 52.365370, longitude −108.875710), SK.

### Canola Genotypes

Sixteen diverse *B. napus* genotypes varying in seed color, glucosinolate, erucic acid, and fiber contents (Table 1) were used in this study to represent genetically diverse germplasm. Five of the *B. napus* lines were breeding lines from the Agriculture and Agri-Food Canada (AAFC) canola breeding program, whereas the other 11 lines were selected from a larger diversity collection comprised of accessions representing the variation found across spring *B. napus* housed within various germplasm collections. A genetic similarity matrix for the 16 *B. napus* genotypes was generated based on single nucleotide polymorphisms (SNPs) across the genome determined using the Brassica 60K Illumina Infinium SNP array (Clarke et al., 2016). Two of the genotypes (Table 1) used in this study have alternate names, NAM-48 or DH27298 and NAM-94 or YN04-C1213sp013.

**TABLE 1:** Sixteen diverse *Brassica napus* genotypes selected for the rhizosphere microbial analysis and their seed quality traits.

***B. napus* Line**	**Origin**	**Seed color**	**Acid detergent Lignin (% of seed)**	**Seed glucosinolates (μmol)**	**Seed erucic acid (% Oil)**
NAM-0^a^	Canada	Black	3.8	8.8	0.44
NAM-13^b^	Europe	Black	7.5	9.5	0.26
NAM-14^b^	Europe	Black	3.2	91	37.81
NAM-17^a^	Canada	Black	3.7	11.3	0.23
NAM-23^b^	North Korea	Black	5.8	10.4	1.1
NAM-30^b^	Europe	Black	8.7	8.6	0.35
NAM-32^b^	South Korea	Black	6.6	114.4	0.18
NAM-37^b^	Australia	Black	6.9	49.9	0.32
NAM-43^b^	South Asia	Black	6.1	92.7	10.14
NAM-46^b^	South Korea	Black	4.5	103.5	47.06
NAM-48^a^	Canada	Yellow	na	na	Na
NAM-5^b^	South Asia	Black	4.2	62.1	9.75
NAM-72^a^	Canada	Yellow	0.8	9.9	0.08
NAM-76^b^	Canada	Black	6.6	14.3	2.18
NAM-79^b^	South Asia	Black	na	na	Na
NAM-94^a^	Canada	Yellow	3.7	119.9	40.08

*^*a*^AAFC Canola breeding lines; ^*b*^diverse annual *Brassica napus* accessions.*

### Experimental Design

The field design in 2016 was a randomized complete block design with three replicates. The three blocks each were comprised of two strips of eight 2 m by 6 m plots. The 16 *B. napus* genotypes were randomly assigned to plots and seeded on May 27, 2016. *B. napus* seeds were pre-treated with HELIX XTra^®^ (active ingredients: Thiamethoxam, difenoconazole, metalaxyl-M, fludioxonil, and sedaxane) (Syngenta Canada Inc., Guelph, ON, United States) and were seeded at a rate of 100 seeds m^–2^ with HELIX-treated corn grits. Roots with attached rhizosphere and bulk soil were collected weekly for 10 weeks starting 18 days after seeding (DAS) and ending 81 DAS. The corresponding phenotypic stages for the 2016 sampling weeks were 2–3 leaf stage (Week 1, 18 DAS), 4–5 leaf stage (Week 2, 25 DAS), 6–9 leaf stage (Week 3, 32 DAS), flowering (Weeks 4–7, 39–70 DAS), maturity (Weeks 8 and 9, 67 and 74 DAS), and harvest (Week 10, 81 DAS). Plants were harvested at maturation to determine seed yield (g) for the entire plot. Each plot-level sample was comprised of three composited subsamples. A subsample was an individual canola plant taken at random from within a plot. For each subsample, bulk and rhizosphere soils were separated, with rhizosphere soil defined as soil that remained attached to roots after shaking to dislodge the loosely attached (bulk) soil (Supplementary Figure S1). Roots of each sub-sample with the adhered rhizosphere soil were handled aseptically, cut and placed together in an Erlenmeyer flask with 100 mL of buffer (0.05 M NaCl), and shaken (Innova 2100 Platform Shaker at 180 r/min) for 15 min. Following shaking, roots were removed from the flask, and the buffer and rhizosphere soil were transferred into two 50 mL centrifuge tubes. Tubes containing the sample mixtures were then centrifuged at 5000 r/min for 15 min at room temperature. The supernatant was decanted and pellets of the rhizosphere soil were subsampled into two 1.5 mL Eppendorf tubes and stored frozen at −80°C.

In 2017, each of the three sites were established following a similar experimental design and sampling protocol to 2016. Seeding of the 16 diverse *B. napus* genotypes was done on May 29 at Saskatoon and May 19 at Melfort. At Scott, delayed seeding date of June 20 was due to re-seeding after hail damage. Rhizosphere soil samples were collected three times in 2017 at the 6–9 leaf stage (Week 3), mid-flowering (Week 6), and maturity (Week 9). Plants were harvested at maturation to determine seed yield (g) for the entire plot.

### DNA Extraction, Amplification, and Sequencing

Rhizosphere soil pellets were removed from the −80°C freezer and 0.1 g was transferred into 96-well DNA extraction plates. DNA was extracted using the DNeasy PowerSoil Kit (Qiagen, catalogue number 12955-4) following the recommended standard procedure. The extracted DNA quantity was determined using a standard Qubit protocol (Thermo Fisher Scientific, Waltham, MA, United States). 16S rRNA genes were amplified using the primer set 342F-806R with Illumina sequencing adapters (342F: 5′-ACA CTG ACG ACA TGG TTC TAC ACT ACG GGG GGC AGC AG-3′ and 806R: 5′-TAC GGT AGC AGA GAC TTG GTC TGG ACT ACC GGG GTA TCT-3′) (Mori et al., 2014). Amplified PCR products were then sent to the Innovation Centre at Genome Quebec for amplicon barcoding, normalization, library QC, and subsequently sequenced on an Illumina MiSeq. Sequencing of the 2017 samples was done at the University of Saskatchewan sequencing facility following the same procedure using an Illumina MiSeq platform. A total of 887 samples were analyzed. Replicates for DNA extraction were individually sequenced. To compare absolute abundance of bacterial 16S rRNA genes across samples, a known amount of a bacterial species not expected to be present in soil (0.3 ng μL^–1^ of *Aliivibrio fisheri*) was spiked into each sample as an internal standard (Smets et al., 2016).

### Sequence Data Processing and Statistical Analysis

Adaptors and primers were trimmed from demultiplexed paired reads using Cutadapt version 2.1 (Martin, 2011). The Cutadapt processed reads were further processed using qiime2 version 2019.1 (Bolyen et al., 2018) DADA2 plugin (Callahan et al., 2016) to filter low-quality and chimera errors and generate a final amplicon sequence variants table and corresponding taxonomic table. DADA2 pipeline is designed to resolve exact biological sequences from Illumina sequence data and does not involve sequence clustering. Following Knight et al. (2018), exact sequence variant approach was used. Oligotyping (Eren et al., 2013) improves upon traditional operational taxonomic unit (OTU) picking, by including position-specific information from 16S rRNA sequencing to identify subtle nucleotide variation and by discriminating between closely related but distinct taxa. The sequences were mapped at 99% sequence identity to an optimized version of the Greengenes reference database (version 13.8) containing the target V3–V5 16S region to determine taxonomies at seven different levels.

The amplicon sequence variant table was further processed to: (1) remove 16S rRNA gene sequences identified as chloroplast, mitochondria, or archaea, (2) adjust sequence counts to account for differences in sequencing depth based on the internal standard (*A. fisheri* counts), and (3) remove *A. fisheri* sequences. We rounded *A. fisheri* adjusted values to the nearest integer prior to analysis with negative binomial generalized linear models (GLMs) which require count data. All other summary statistics and analyses were completed using the unrounded values unless stated. Statistical analyses were done in R (version 3.6.0, R Core Team, 2019) using Phyloseq (version 1.22.3) (McMurdie and Holmes, 2013), microbiome (version 1.5.28) (Leo and Sudarshan, 2017), and their associated dependencies.

The *B. napus* core microbiota, or the set of amplicon sequence variants detected in 50–100% of the samples with a relative abundance threshold value above 0.01%, was identified using the core function in microbiome R package version 1.5.28 (Leo and Sudarshan, 2017) using the whole dataset from Saskatoon in 2016. The dataset was then grouped into three broad phenological stages: vegetative (weeks 1–3), flowering (weeks 4–7), and maturity (weeks 8–10). Core bacterial taxa with at least 75% prevalence were determined within each phenological category and were compared with the core taxa identified for the whole dataset to determine if there were patterns in how the core taxa were recruited. Core bacterial taxa with at least 75% prevalence were determined for each of the three sampling sites in 2017, and then compared between sites and to the core bacterial taxa from the single site 2016 experiment.

Prior to α-diversity calculations and permutational multivariate analysis of variance (PERMANOVA) test (Anderson, 2001), the amplicon sequence variant table was normalized using the edgeR “edgernorm” method in R package microbiomeSeq (Ssekagiri et al., 2017). The α-diversity indices (Richness, Pielou’s evenness) calculations, pairwise ANOVA of the diversity measures between canola genotypes as well as between sampling weeks were done using microbiomeSeq and microbiome (Leo and Sudarshan, 2017) packages. Bray–Curtis distance for each pair of samples was calculated and PEMANOVA and homogeneity of dispersion test were run using the “adonis” and “betadisper” functions in R package Vegan (Oksanen et al., 2019). The 2016 10-week dataset and its subsets vegetative, flowering, and maturity stages were used for α-diversity and vegetative, flowering, vegetative and flowering combined, and flowering and maturity stages combined subsets for PERMANOVA analysis.

We calculated the mean Bray–Curtis distance between canola genotypes by averaging the paired sample values of each group. Person’s correlation coefficient between plant genetic distance and the mean microbial Bray–Curtis distance among the genotypes were then calculated. The datasets that showed difference in microbial community structure among canola genotypes (PERMANOVA test) were used for this analysis.

We considered the diversity indexes calculated as phenotypic records and hence estimated their broad-sense heritability (*h*^2^) separately for flowering and whole 2016 datasets. To estimate *h*^2^, first variance components of each variable (richness, evenness, and other calculated indices) were generated by fitting a linear mixed effects model using restricted maximum likelihood (REML) with the lmer function of the lme4 R package version 1.1.21 (Bates et al., 2019). The two models where: *response (whole data)* = *canola genotype* + *Rep (Week)* + *canola X week* + *residual*, and *response (flowering)* = *canola* + *canola X week* + *residual*. Due to model over parameterization and singular fit “Rep (week)” (replication nested in sampling week) from whole data model and “canola X week” (canola sampling week interaction) from the flowering stage model were dropped in the final model. The *h*^2^ decreased by a maximum of only 0.01 in the simpler models. The proportion of variance explained by canola genotype (*h*^2^) was calculated using the formula *h*^2^ = σ *_*G*_*^2^/(σ*_*G*_*^2^ + (σ*_*G*_*_x_ *_*W*_* ^2^)/*w* + σ*e*^2^/*rw*) for the whole 2016 data, or *h*^2^ = σ*_*G*_*^2^/(σ*_*G*_*^2^ + σ*e*^2^/*rw*) for flowering stage data, where σ*_*G*_*^2^ is genetic variance, σ*_*G*_*_x_ *_*W*_*^2^ is the variance of genotype by sampling week interactions, σ*e*^2^ is residual variance, *r* is the number of replications in each sampling week, and *w* is the number of sampling weeks.

Bacterial genera that were differentially abundant between each of the 15 *B. napus* genotypes and a reference genotype (NAM-0) were identified using negative binomial models fitted in GLM framework using the edgeR package version 3.8 (Robinson et al., 2010). The 2016 Saskatoon dataset was first agglomerated to the lowest identified taxonomic rank (genus), resulting in a total of 558 genera. First, Upperquartile (Bullard et al., 2010) normalization was used to align the upper quartiles of the count per million within the libraries. The goal was to identify bacterial taxa that were differentially abundant between the reference genotype and each of the 15 remaining *B. napus* genotypes. Hence, the design matrix contained *B. napus* genotype with the intercept set as NAM-0, and a significant coefficient for a given genotype identified significant abundance differences from NAM-0. Dispersion estimates were subsequently calculated using estimateDisp for the given design matrix. A quasi-negative-binomial-GLM was fit with the glmQLFit function, and significance tested with the glmQLFtest function. A 1% false discovery rate (FDR; Benjamin and Hochberg adjusted *p*-value) was used. R scripts for the core bacteria, heritability and differential abundance analysis are provided (Supplementary Data Sheets S1–S3).

## Results

### Rhizosphere Taxonomic Characterization

A total of 1,571,433,759 reads (ranging from 5925 to 354,234,808 per sample) across 477 samples (Supplementary Table S1) were present in the dataset. We detected 32 named bacterial phyla (Supplementary Table S2 and Supplementary Figure S2), approximately a third of the currently named phyla (Hug et al., 2016). Proteobacteria, Acidobacteria, Actinobacteria, and Chloroflexi were the most abundant phyla, present in 69.4, 13.8, 12.2, and 4.6% of the samples, respectively (Supplementary Tables S3, S4). Four named classes were identified from phylum Proteobacteria which included the common Gammaproteobacteria, Alphaproteobacteria, Betaproteobacteria, and Deltaproteobacteria, 15 classes from Acidobacteria, and 10 from Actinobacteria.

### Single Site 2-Year *B. napus* Rhizosphere Core Microbiome

Six bacterial genera were identified as core at a 75% prevalence threshold; at 50, 60, 65, and 70% prevalence thresholds the number of core genera were 32, 15, 11, and 8, respectively. The *B. napus* core microbiome primarily included Proteobacteria and Actinobacteria. *Arthrobacter* (Actinobacteria) was found in 96% of the samples, *Bradyrhizobium* (Proteobacteria) in 95%, Stenotrophomonas (Proteobacteria), *Skermanella* (Proteobacteria), and one *unclassified* Acidobacteria and one unclassified Actinobacteria were present in 75% of the samples (Table 2 and Supplementary Table S5). Three of the four named core taxa in 2016 were consistently observed in the 2017 experiment. A total of 11 core bacterial taxa were identified at 75% in the 2017 experiment (Table 3 and Supplementary Table S5).

**TABLE 2:** Overall and growth stage-related *B. napus* core bacterial taxa identified at Saskatoon 2016 experiment.

**Taxonomy**						**Core in**			
	
**Phylum**	**Class**	**Order**	**Family**	**Genus**	**Species**	**t**	**v**	**f**	**m**
Acidobacteria	Chloracidobacteria	RB41	Ellin6075	*Unclassified*		Y	Y	Y	Y
Actinobacteria	Actinobacteria	Actinomycetales	Micrococcaceae	*Arthrobacter*		Y	Y	Y	Y
				*Arthrobacter*				Y	Y
				*Arthrobacter*				Y	Y
			Microbacteriaceae	*Agromyces*					Y
	Thermoleophilia	Gaiellales	Gaiellaceae	*Unclassified*		Y	Y	Y	
Proteobacteria	Alphaproteobacteria	Rhodospirillales	Rhodospirillaceae	*Skermanella*		Y		Y	Y
		Rhizobiales	Bradyrhizobiaceae	*Bradyrhizobium*		Y	Y	Y	Y
	Gammaproteobacteria	Pseudomonadales	Moraxellaceae	*Acinetobacter*	*rhizosphaerae*			Y	
		Xanthomonadales	Xanthomonadaceae	*Stenotrophomonas*	*retroflexus*	Y		Y	Y

*“Y” represents “yes” for the four rightmost columns under “Core in.” t, overall or total; v, vegetative; f, flowering; and m, maturity growth stages. Repeated genus name indicates the presence of amplicon sequence variant. Please see Supplementary Table S5 for full table showing their corresponding prevalence and relative abundance.*

**TABLE 3:** Cross-year *B. napus* core bacterial taxa identified in at least 75% of the samples at Saskatoon site.

**Taxonomy**						**Core in**	
	
**Phylum**	**Class**	**Order**	**Family**	**Genus**	**Species**	**2016**	**2017**
Acidobacteria	Chloracidobacteria	RB41	Ellin6075	*Unclassified*		Y	
Actinobacteria	Actinobacteria	Actinomycetales	Micrococcaceae	*Arthrobacter*		Y	Y
			Propionibacteriaceae	*Microlunatus*			Y
	Thermoleophilia	Gaiellales	Gaiellaceae	*Unclassified*		Y	
Bacteroidetes	Sphingobacteriia	Sphingobacteriales	Sphingobacteriaceae	*Pedobacter*			Y
				*Unclassified*			Y
Proteobacteria	Gammaproteobacteria	Enterobacteriales	Enterobacteriaceae	*Erwinia*			Y (2)^∗^
		Pseudomonadales	Pseudomonadaceae	*Pseudomonas*			Y
	Alphaproteobacteria	Rhizobiales	Bradyrhizobiaceae	*Bradyrhizobium*		Y	Y
		Rhodospirillales	Rhodospirillaceae	*Skermanella*		Y	
		Sphingomonadales	Sphingomonadaceae	*Kaistobacter*			Y
	Gammaproteobacteria	Xanthomonadales	Xanthomonadaceae	*Stenotrophomonas*	*retroflexus*	Y	Y
				*Stenotrophomonas*	*maltophilia*		Y

*Weekly sampling for 10 weeks in 2016 and at three growth stages in 2017. “2016” and “2017” represent the study years. The number in bracket indicates the number of amplicon sequence variants for that genus. Please see Supplementary Table S5 for full table showing their corresponding prevalence and relative abundance. ^∗^There were two amplicon sequence variants for that genus.*

Core genera associated with each growth stage were generally part of the overall core taxa at the 75% prevalence threshold. *Arthrobacter*, *Bradyrhizobium*, and an unclassified Acidobacteria in the class Ellin6075 were present in all growth stages, while two variants of *Arthrobacter*, *Stenotrophomonas, and Skermanella* joined the core taxa at flowering. One unclassified Gaiellaceae (Actinobacteria) is observed only at the vegetative and flowering stages. *Acinetobacter* a Gammaproteobacteria in family Moraxellaceae was only present during flowering while *Agromyces* an Actinobacteria in family Microbacteriaceae was only present in the maturity stage (Table 2).

### 2017 Three-Site *B. napus* Rhizosphere Core Microbiome

The 2017 cross-site core microbiome closely resembled the 2016 core microbiome with 11, 21, and 7 core bacterial genera at 75% prevalence at Saskatoon, Melfort, and Scott sites, respectively. The cross-site *B. napus* core microbiome primarily included Proteobacteria and Actinobacteria. *Arthrobacter*, *Erwinia* spp. (two variants), *Kaistobacter*, *Pedobacter*, and *Stenotrophomonas* (*Stenotrophomonas retroflexus*) were cross-site conserved. *Bradyrhizobium* and *Microlunatus* were conserved at two sites (Saskatoon and Melfort) (Table 4 and Supplementary Table S5). *Arthrobacter* was the most prevalent core taxa at Saskatoon (both years) and Melfort and *Stenotrophomonas* was most prevalent at Scott.

**TABLE 4:** *Brassica napus* core bacterial taxa identified in at least 75% of the samples at Saskatoon, Melfort, and Scot sites.

**Taxonomy**						**Core at**		

**Phylum**	**Class**	**Order**	**Family**	**Genus**	**Species**	**SA**	**ME**	**SC**
Actinobacteria	Actinobacteria	Actinomycetales	Micrococcaceae	*Arthrobacter*		Y	Y (2)^∗^	Y
			Propionibacteriaceae	*Microlunatus*		Y	Y	
			Nocardiaceae	*Rhodococcus*			Y	
		Micrococcales	unclassified	*Unclassified*			Y	
	Thermoleophilia	Gaiellales	Gaiellaceae	*Unclassified*			Y (2)^∗^	
Bacteroidetes	Sphingobacteriia	Sphingobacteriales	Sphingobacteriaceae	*Pedobacter*		Y	Y	Y
				*Unclassified*		Y	Y	
				*Mucilaginibacter*	*gossypii*		Y	
Firmicutes	Bacilli	Bacillales	Bacillaceae	*Bacillus*	*longiquaesitum*			Y
Gemmatimonadetes	Gemmatimonadetes	Unclassified	Unclassified	*Unclassified*			Y	
Planctomycetes	Phycisphaerae	WD2101	Unclassified	*Unclassified*			Y	
Proteobacteria	Alphaproteobacteria	Sphingomonadales	Sphingomonadaceae	*Kaistobacter*		Y	Y	Y
		Rhizobiales	Bradyrhizobiaceae	*Bradyrhizobium*		Y	Y	
			Hyphomicrobiaceae	*Rhodoplanes*			Y	
		Caulobacterales	Caulobacteraceae	*Caulobacter*			Y	
	Betaproteobacteria	Burkholderiales	Comamonadaceae	*Variovorax*	*paradoxus*		Y	
		SC-I-84	Unclassified	*Unclassified*			Y	
	Gammaproteobacteria	Pseudomonadales	Pseudomonadaceae	*Pseudomonas*		Y		
		Xanthomonadales	Xanthomonadaceae	*Stenotrophomonas*	*retroflexus*	Y	Y	Y
				*Stenotrophomonas*	*maltophilia*	Y		
		Enterobacteriales	Enterobacteriaceae	*Erwinia*		Y (2)^∗^	Y (2)^∗^	Y (2)^∗^

*“Y” represents “yes” for the three rightmost columns under “Core at.” SA, Saskatoon; ME, Melfort; and SC, Scott sites. The numbers in bracket indicate the number of amplicon sequence variants for that genus. Please see Supplementary Table S5 for full table showing their corresponding prevalence and relative abundance. ^∗^There were two amplicon sequence variants for that genus.*

### Genotype Variations in Alpha Diversity Measures

**FIGURE 1:**
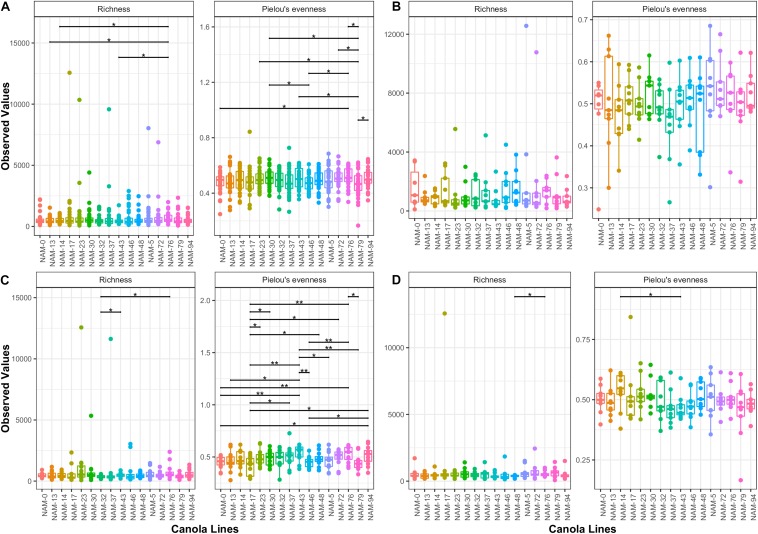
Variability in alpha diversity measures (richness and evenness) among canola genotypes. Bars connect significantly different canola genotype pairs and significance level is indicated with an asterisk (^∗^0.05, ^∗∗^0.01). Figures present alpha diversity comparisons based on **(A)** Whole 2016 dataset, **(B)** vegetative, **(C)** flowering, and **(D)** maturity stages.

Alpha diversity measures (richness and Pielou’s evenness) varied among the canola genotypes. More variability is observed in Pielou’s evenness. Flowering stage followed by whole data analysis resulted in the highest variability among genotypes (Figure 1). No significant difference among genotypes was observed at vegetative stage and only two pairs of genotypes (NAM-48 – NAM-76 and NAM-14 – NAM-43) showed significant difference in richness and Pielou’s evenness at maturity.

### Microbial Patterns Among *B. napus* Genotypes

The PERMANOVA and betadisper analyses were used to test whether the rhizosphere bacterial community structure differed among *B. napus* genotypes. The PERMANOVA partitions the variability between factors based on the Bray–Curtis dissimilarity matrix. To check the contribution of growth stage at which samples were collected, we used the complete 2016 dataset and its subsets. Statistically significant differences were observed in the rhizosphere microbiome among *B. napus* genotypes when using the complete (*F* = 1.23, *R*^2^ = 0.04, *p* < 0.001), flowering (*F* = 1.12, *R*^2^ = 0.09, *p* < 0.05), vegetative and flowering combined (*F* = 1.14, *R*^2^ = 0.05, *p* < 0.05), and flowering and maturity combined (*F* = 1.17, *R*^2^ = 0.05, *p* < 0.01) datasets. The test for homogeneity of multivariate dispersions was not significant indicating that canola genotypes have similar dispersion and we can trust the significant variation observed. We did not observe significant variation in the rhizosphere microbiome among *B. napus* genotypes at the vegetative and maturity stages.

### Relationship Between *B. napus* Genotypes and Rhizosphere Microbial Composition

**FIGURE 2:**
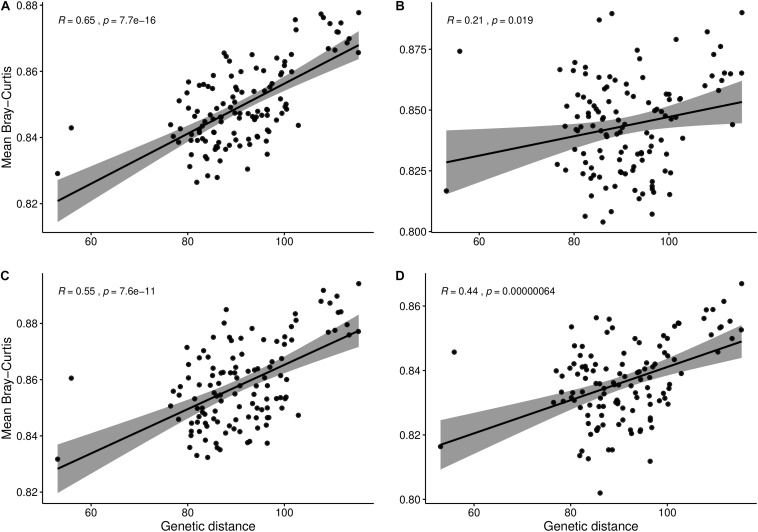
Correlation between mean microbial Bray–Curtis distance and plant genetic distance among canola genotypes. Change in correlation when considering the **(A)** whole 2016 dataset, **(B)** flowering stage, **(C)** vegetative and flowering stages combined, and **(D)** flowering and maturity stages combined.

To check whether the composition of the rhizosphere microbiota could be correlated to the genetic relatedness among the *B. napus* genotypes, we computed mean microbial Bray–Curtis distances and correlated those with plant genetic distance among the genotypes. In addition, to infer if the correlation is affected by the growth stage of *B. napus*, we did correlations using datasets from flowering, vegetative and flowering combined, and flowering and maturity stages combined. The vegetative stage was omitted since we did not find significant variation during the PERMANOVA analysis. The significant (*p* < 0.001) and highest (*R* = 0.65) correlation was observed when using the dataset across the whole growing season followed by in the vegetative and flowering stage combined (*p* < 0.001, *R* = 0.55), and flowering and maturity stage combined datasets (*p* < 0.001, *R* = 0.44), respectively (Figure 2). At flowering the least but significant correlation was observed (*p* < 0.05, *R* = 0.21).

### Heritability of Microbial Attributes

The heritability estimates for the alpha diversity measures were higher at flowering than for the whole growing season (Tables 5, 6). At flowering, diversity inverse Simpson and evenness Simpson were with the highest heritability (*h*^2^ = 0.59) followed by diversity Shannon and evenness Pielou (*h*^2^ = 0.51). Diversity gini Simpson (*h*^2^ = 0.37) followed by diversity inverse Simpson and evenness Simpson (*h*^2^ = 0.25) were with the highest heritability estimates for the whole growing season.

**TABLE 5:** Broad-sense heritability of alpha diversity traits (diversity_inverse_simpson, diversity_gini_simpson, diversity_shannon, evenness_pielou, evenness_simpson) using the full 10 weeks dataset of 2016.

**Trait (complete)**	**Variance component**			**Heritability (*h*^2^)**
		
	**Genotype**	**Genotype × week**	**Residual**	
Diversity_inverse_simpson	46.79	204.49	3688.61	0.25
Diversity_gini_simpson	0.00002678	0.00002116	0.00132184	0.37
Diversity_shannon	0.003671	0.01906	0.542428	0.16
Evenness_pielou	0.0000412	0.0002139	0.0060884	0.16
Evenness_simpson	3.065E-07	1.1227E-06	2.33607E-05	0.25
				

**TABLE 6:** Broad-sense heritability of alpha diversity traits (diversity_inverse_simpson, diversity_gini_simpson, diversity_shannon, evenness_pielou, evenness_simpson) using the flowering stage dataset of 2016 experiment.

**Trait (F)**	**Variance component**		**Heritability (*h*^2^)**
		
	**Genotype**	**Residual**	
Diversity_inverse_simpson	403.1	3366.9	0.59
Diversity_gini_simpson	0.00005502	0.00114562	0.37
Diversity_shannon	0.0489	0.5645	0.51
Evenness_pielou	0.0005489	0.0063357	0.51
Evenness_simpson	2.552E-06	2.1319E-05	0.59

### Differentially Abundant Bacterial Genera

**FIGURE 3:**
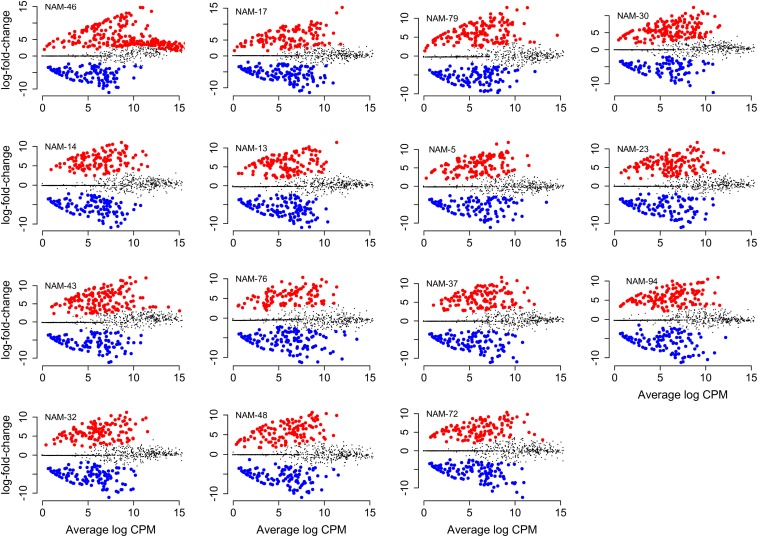
Differentially abundant bacterial genera compared with the reference genotype (NAM-0). Change in absolute abundance in *B. napus* genotypes (log fold change) is shown against average abundance in count per million (CPM). Red dot indicates significantly differentially more and blue less abundant taxa (FDR < 0.01). The non-significant genera are indicated in gray. Names of *B. napus* genotypes are indicated at the top left corner of individual plots. Plots are arranged left to right from genotype with the highest to the lowest number of differentially abundant genera compared with NAM-0.

Of the total 558 genera, 81 bacterial genera were significantly (*p* < 0.01) more and 71 genera were similarly less abundant in at least one *B. napus* genotype relative to the reference genotype (NAM-0). With the exception of genotypes NAM-14, NAM-30, NAM-32, NAM-43, and NAM-46, there was an observed general trend toward differentially less abundant taxa per genotype (Table 7 and Figure 3). The most genetically distinct genotype (NAM-46) had the greatest number of differentially highly abundant taxa; however, there was not a clear pattern linking plant genetic distance across the diversity panel with number of differentially abundant bacterial taxa (Table 7 and Supplementary Figure S3).

**TABLE 7:** Number of differentially less and more abundant bacterial genera at 1% false discovery rate in 15 *B. napus* lines compared with the reference NAM-0.

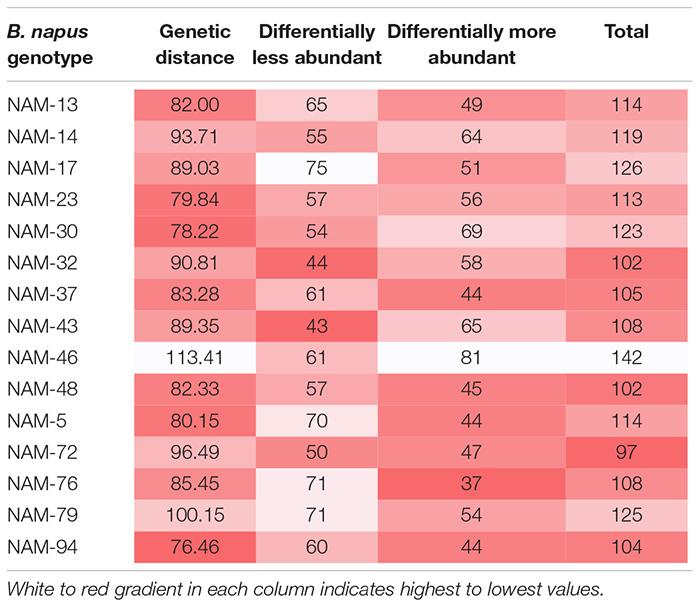

A total of 29 bacterial phyla (17–23 phyla per genotype) were represented among the differentially abundant bacterial genera. The majority were Proteobacteria and Actinobacteria, together accounting for 52–61% of differentially abundant genera in each *B. napus* genotype. Phylum FBP, Tenericutes, and BRC1were unique phyla observed only in NAM-13, NAM-14, and NAM-48, respectively.

Across the 15 genotypes, there were 67 genotype-specific (Table 8) differentially abundant bacterial genera of which 56 (84%) were differentially more abundant and 11 (16%) less abundant compared with the reference NAM-0. Per *B. napus* genotype, the number of unique genera ranged from one [NAM-23, ∼2% (per total number of genotype-specific genera)] to 11 (NAM-46, 16%). In NAM-76, all unique genera were differentially less abundant whereas in NAM-5, NAM-13, NAM-14, NAM-23, NAM-32, NAM-46, and NAM-94, the unique genera were differentially more abundant.

**TABLE 8:** Differentially abundant named bacterial genera unique to each *B. napus* genotypes in 2016 and their traits or plant beneficial roles.

**Canola lines**	**Genus**	**Phylum**	**Trait/role**	**References**
NAM-13	*Saccharothrix*	Actinobacteria	Plant growth promotion	Compant and Mathieu, 2013
	*Procabacter*	Proteobacteria	Grazed by free living bacteria	Schmitz-Esser et al., 2008
	*Crocinitomix*	Bacteroidetes	Correlation with soil dehydrogenase	Ye, 2016
	*Aspromonas*	Proteobacteria	–	–
NAM-14	*Rubricoccus*	Bacteroidetes	Salt tolerant	Rath, 2017
	*Veillonella*	Firmicutes	Most abundant at flowering	Xiao et al., 2017
	*Anaerococcus*	Firmicutes	Associated with Root Lesion Nematodes	Elhady et al., 2017
	*Sporosarcina*	Firmicutes	Plant growth promotion	Janarthine and Eganathan, 2012
	*Polaromonas*	Proteobacteria	Desulfonation	Schmalenberger et al., 2008
	*Neisseria*	Proteobacteria	Present in disease suppressive soils	Almario et al., 2013; Adam et al., 2014
	*Granulicatella*	Firmicutes	–	–
	*Asteroleplasma*	Tenericutes	–	–
	*Roseateles*	Proteobacteria	Plant growth promotion	Muthukumarasamy et al., 2017
NAM-17	*Citrobacter*	Proteobacteria	Biocontrol	Abiala et al., 2015
	*Pelosinus*	Firmicutes	Methanogenic	Dai et al., 2016
	*Emticicia*	Bacteroidetes	Modulating flowering; denitrification	Lu et al., 2018
	*Leifsonia*	Actinobacteria	N_2_-fixing	Rilling et al., 2018
	*Aeromicrobium*	Actinobacteria	Plant growth promotion	Yadav et al., 2015
NAM-23	*Sediminibacterium*	Bacteroidetes	Negatively associated with soil Ph	Xiao et al., 2017
NAM-30	*Shinella*	Proteobacteria	Nitrate reduction	Lee et al., 2011
	*Curtobacterium*	Actinobacteria	Bacterial wilt; IAA, metal accumulation	Hsieh et al., 2005; Janssen et al., 2015
	*Turicibacter*	Firmicutes	–	–
	*Aerococcus*	Firmicutes	Antimycobacterial	Zahir et al., 2011
	*Spirillospora*	Actinobacteria	Source of antibiotics	Hacène et al., 2000
	*Afipia*	Proteobacteria	–	–
	*Gardnerella*	Actinobacteria	–	–
	*Paenibacillus*	Firmicutes	Plant growth promotion	Grady et al., 2016
	*Delftia*	Proteobacteria	Phosphate solubilizing	Chen et al., 2006
	*Caldilinea*	Chloroflexi	–	–
NAM-32	*Moellerella*	Proteobacteria	–	–
	*Marmoricola*	Actinobacteria	–	–
	*Coprococcus*	Firmicutes	–	–
NAM-37	*Actinobacillus*	Proteobacteria	–	–
	*Pirellula*	Planctomycetes	–	–
	*Haliea*	Proteobacteria	–	–
	*Mesorhizobium*	Proteobacteria	Salt tolerant	Jha et al., 2012
NAM-43	*Anaeromyxobacter*	Proteobacteria	Methanotroph	Yoneyama et al., 2017
	*Actinoallomurus*	Actinobacteria	Plant defense	Visioli et al., 2018
	*Uliginosibacterium*	Proteobacteria	–	–
	*Ilumatobacter*	Actinobacteria	–	–
	*Vibrio*	Proteobacteria	Phosphate solubilizing	Vazquez et al., 2000
NAM-46	*Enterococcus*	Firmicutes	–	–
	*Klebsiella*	Proteobacteria	Plant growth promotion	Sachdev et al., 2009
	*Hydrogenophaga*	Proteobacteria	Phytoremediation	Hou et al., 2015
	*Knoellia*	Actinobacteria	–	–
	*Chthoniobacter*	Verrucomicrobia	–	–
	*Naxibacter*	Proteobacteria	Arsenic resistant	Huang et al., 2010
	*Acidovorax*	Proteobacteria	Antagonist to *Fusarium solani*	Fan et al., 2016
	*Dyadobacter*	Bacteroidetes	Biocontrol	Ciancio et al., 2016
	*Mycoplana*	Proteobacteria	Plant growth promotion	Egamberdiyeva and Höflich, 2003
	*Agrobacterium*	Proteobacteria	Plant growth promotion	Bertrand et al., 2001
	*Stenotrophomonas*	Proteobacteria	Plant growth promotion	Wolf et al., 2002; Lay et al., 2018
NAM-48	*Weissella*	Firmicutes	Antimycobacterial	Fhoula et al., 2013
	*Amaricoccus*	Proteobacteria	–	–
	*Caulobacter*	Proteobacteria	Plant growth promotion	Yang et al., 2019
NAM-5	*Frigoribacterium*	Actinobacteria	Zn solubilizing	Costerousse et al., 2018
	*Planomicrobium*	Firmicutes	Nutrient mobilization	Shahid et al., 2018
	*Sporichthya*	Actinobacteria	Improved soil chemical property	Zhang et al., 2019
NAM-76	*Candidatus Xiphinematobacter*	Verrucomicrobia	Nematode endosymbiont	Mobasseri et al., 2019
	*Streptosporangium*	Actinobacteria	Biocontrol	Boukaya et al., 2018
NAM-79	*Anaerolinea*	Chloroflexi	–	–
	*Serratia*	Proteobacteria	Biocontrol; plant growth promotion	Lay et al., 2018; Purkayastha et al., 2018
	*Catellatospora*	Actinobacteria	–	–
	*Afifella*	Proteobacteria	–	–
NAM-94	*Segetibacter*	Bacteroidetes	–	–
	*Thiobacillus*	Proteobacteria	Sulfur oxidizing	Huber et al., 2016
	*Wohlfahrtiimonas*	Proteobacteria	Arsenic metabolizing	Sanyal et al., 2016

*Most of these genera are probably for the first time being reported in the rhizosphere of canola.*

We ranked differentially abundant bacteria based on abundance changes relative to the reference line [log fold change (logFC)] to identify bacteria with the strongest population responses to each *B. napus* genotype. Responses in both positive (more abundant) and negative (less abundant) directions were observed. For example, in the genetically distinct genotype NAM-46 there were more genera with strong positive responses, this also seemed the case for most lines (Supplementary Figure S4). The full list of the significantly differentially abundant bacterial genera within each genotype together with their corresponding taxonomic classification and values for logFC, log count per million (logCPM), *p*-value, and FDR are provided in Supplementary Table S6.

## Discussion

### *B. napus* Genotype Regulated Changes in Rhizosphere Bacteria

Here we show fine-scale regulation of the rhizosphere bacterial community by *B. napus* host genotypes as a total of 67 differentially abundant genera were genotype-specific. This suggests an extensive and selective control by *B. napus* genotype on associated rhizosphere bacterial genera. Given these controls are genetically based, they may represent potential breeding targets if the associated bacterial shown to be positively associated with yield or positive traits in subsequent work. The most genetically distinct *B. napus* genotype (NAM-46) contributed a greater proportion (∼16%) of the genotype specific differentially abundant taxa, and almost all of its top 20 differentially abundant genera based on logFC were differentially more abundant (Supplementary Figure S4). This suggests that genetic variability among genotypes might be directed toward investing in recruiting taxa rather than efforts toward exclusion (reduction in abundance). The phyla that were mainly responsive to *B. napus* line were Proteobacteria and Actinobacteria; similar responsiveness of these phyla to dynamic changes in bacterial community has been reported (Yang et al., 2017).

Several potentially important beneficial *B. napus* genotype–bacterial interrelationships were evident among the differentially abundant taxa. Differentially abundant bacteria with potential benefits include *Cellulosimicrobium*, *Enterobacter*, *Klebsiella*, *Modestobacter*, *Neisseria*, *Peredibacter*, *Pseudomonas*, *Serratia*, and *Streptosporangium*. These bacteria have been reported to have roles in the biological control of diseases, including production of antibiotics, association with disease suppressive soils, anti-fungal properties, and biocontrol mechanisms. *Cellulosimicrobium* spp., for example, inhibit growth of the pathogenic fungi *Botryis cinerea*, *Fusarium oxysporum*, and *Verticillium dahlia* in barley (Nabti et al., 2014). *Enterobacter* spp. produce complex compounds that are antagonistic toward many fungal pathogens and also have antibiotic properties (Chernin et al., 1995, 1996). *Neisseria* is associated with disease suppressive soils (Almario et al., 2013; Adam et al., 2014), *Peredibacter* suppresses soil-borne disease (Jaiswal et al., 2017), and *Pseudomonas* induces systemic resistance against pathogens such as the soil-borne *Rhizoctonia solani* (Nandakumar et al., 2001). *Streptosporangium* is an Actinomycete known to produce antimicrobial compounds (Hardoim et al., 2015). Finally, *Serratia* promotes resistance to fungal pathogens in rapeseed (Alström, 2001; Neupane et al., 2013). It can also parasitize pathogenic *Fusarium* and reduces the production of areal hyphae and microconidia (Minerdi et al., 2008).

In addition to disease protection, some of the differentially abundant bacteria have beneficial effects including growth promotion. *Cellulosimicrobium* spp. can stimulate growth of barley seedlings (Nabti et al., 2014), and *Enterobacter* spp. are important plant growth-promoting bacteria (Chernin et al., 1996; Nie et al., 2002). Strains of *Klebsiella* isolated from wheat rhizosphere produce indole acetic acid (IAA) with demonstrated promotion of root growth (Sachdev et al., 2009). *Serratia* is a plant growth-promoting bacteria and is positively correlated with canola yield (Lay et al., 2018) and promotes growth in rapeseed (Alström, 2001; Neupane et al., 2013). Finally, Pseudomonas, a well-studied plant growth-promoting bacterium enhances root elongation of canola and is characterized by its ability to produce phytohormones, IAA, and cytokinin (Pallai et al., 2012).

The potential roles of the differentially abundant bacterial taxa discussed above are limited to genera with functions previously reported in the literature; examination of the uncharacterized and unclassified bacteria associated with high-performing *B. napus* genotypes will likely reveal additional positive plant–microbial interrelationships. Evaluating genotypes with contrasting differential abundance of bacteria that influence traits of interest such as disease resistance and yield would be the next step toward practical applications of *B. napus* microbiome manipulation. The further dissection of the highlighted *B. napus*–bacterial interrelationships is crucial in conceptualizing their use as potential targets for direct or indirect selection within breeding programs.

The observed high correlation between the mean microbial Bray–Curtis distance and plant genetic distance (*R* = 0.65) indicates that overall microbial variation is a good predictor of plant genetic distance. Such observations under field conditions encourage further dissection of plant genetic control on the Canola microbiome. The high variability in species evenness compared with species richness also indicates as there could be direct processes including changes in rhizosphere soil chemistry or other indirect effects by host genotype that alters proportional diversity through changes in evenness without changes in species composition (Wilsey and Potvin, 2000). These potential host-driven changes showed maximum effect during flowering stage with close to no effects during vegetative and maturity stages to grant significant variation in evenness among the genotypes. This suggests that plant genetics-driven changes start to be apparent around flowering stage.

This study is also among the first to describe alpha diversity measures as phenotypic traits in canola and to estimate heritability both considering the whole growing season and at flowering stage. The heritability range for different traits (0.37–0.59) at flowering stages indicates that 37–59% of the variation in alpha diversity measures in our canola genotypes at flowering was due to genetics. Across growth stages 16–37% of the variation in our canola genotypes was either directly or indirectly due to genetics. Diversity is generally associated with resilience to perturbations and disease suppression in rhizosphere, thus diversity could be a valuable trait to consider for sustainable and improved crop production. Maximum heritability values were observed at flowering, again suggesting maximum genetic effects at this growth stage. Therefore, if the alpha diversity measures are to be used as indicator traits, we suggest selections to be made at the flowering stage.

### *B. napus* Rhizosphere Core Microbiome

Microbes that are consistently present across all cultivars and sites are likely to provide critical ecological functions (Shade and Handelsman, 2012). Our cross-site core taxa analysis revealed taxa that were conserved across sites (Table 4). This suggests that *B. napus* genotypes could select relatively conserved core bacterial taxa across a variety of soil types and environments. In contrast to most core microbiome studies, which use samples collected at a single time point, our sampling approach (10 weeks, at least 28 samples per canola genotype) provides insights into how the core members associate with the plant through growth and development stages. Separating the core taxa for each of the three growth stages yielded three key insights into the ecology of the core microbiome. First, there were core members that remained present throughout the development of the crop; second, there were taxa that joined the core membership starting at flowering and continued through to maturity and third, there were taxa that joined the core membership only at a particular growth stage but were not retained in other stages. These findings suggest that efforts to determine the core microbiota of crop species that are limited in the volume and/or timing of sampling should focus on the periods of flowering and/or maturity to get the best overall representation. Additional similar study in a different crop is needed to see if the same phenomenon holds. Cross-site and cross-year stability of core members such as *Arthrobacter*, *Bradyrhizobium*, and *S. retroflexus* indicate their close association with canola.

Core microbiota members with potential beneficial effects include *Arthrobacter*, *Stenotrophomonas*, and *Bradyrhizobium*. *Arthrobacter* can increase canola yield when applied as a bacterial suspension to seeds (Kloepper et al., 1988). *Stenotrophomonas* spp. including *Stenotrophomonas rhizophila* have antagonistic activity against fungal pathogens including *V. dahlia* in oil seed rape (Alström, 2001; Wolf et al., 2002). *Arthrobacter* and *Stenotrophomonas* are likely broadly present in the core microbiome of canola, having been identified in other surveys of the core rhizosphere microbiome of field grown canola (Lay et al., 2018) and in related *Brassica rapa* (Germida et al., 1998; Siciliano and Germida, 1999; Larcher et al., 2008; Croes et al., 2013; Tkacz et al., 2015). Positive correlations between *Arthrobacter* and *Stenotrophomonas* with canola yield (Lay et al., 2018) suggest an important beneficial influence of these bacteria on canola. We observed two variants of *Arthrobacter* joining the core microbiome during flowering, suggesting an important role in the reproductive phase of canola development. Finally, *Bradyrhizobium* shares characteristics with plant growth-promoting rhizobacteria, producing compounds including phytohormones, siderophores, and hydrogen cyanide, and exhibiting antagonistic effects toward many plant pathogenic fungi (Antoun et al., 1998). Core bacteria with such antagonistic effects toward pathogens can be targets to enhance suppressive conditions in the rhizosphere. For instance, a suppressive environment in the rhizosphere can contribute to the failure of invasion and disease development by *Plasmodiophora brassicae*, the causative agent of clubroot disease (Mattey and Dixon, 2015; Dixon, 2017). Focusing on potentially co-evolved core bacterial taxa with both antagonistic potential and a competitive advantage in the *B. napus* rhizosphere could be a strategy to boost the suppressive potential of the rhizosphere to provide a control approach for clubroot or other diseases.

### Harnessing Plant–Microbial Interactions for Canola Breeding

Harnessing the potentially beneficial plant–microbe interactions identified here will require research in a number of directions. Our approach using core-microbiota and differential abundance analysis, correlations between microbial Bray–Curtis distance and plant genetic distance and heritability of alpha diversity measures shows that genotype of *B. napus* may drive very specific associations. Although potentially positive associations are cited throughout literature, testing canola genotype-specific relationships is key to establishing if selecting for microbiome as a trait is feasible. Once specific positive interactions are confirmed in canola, there are several approaches that could be taken to enhance the benefits. For example, one could manipulate the microbiome by breeding plants that have a competitive advantage for particular microbes (Bakker et al., 2012). Our differential abundance analysis identified bacterial taxa that appear selectively favored in each *B. napus* genotypes; closely examining the genetic differences between those genotypes could shed light on the genetic basis for specific plant–microbe relationships. Once a genetic basis for a plant–microbe relationship is identified, breeders can work to move that trait into new lines. Developing microbial consortia that are evolutionarily adapted to the host plant of interest is another approach to harness beneficial microbial functions in agricultural systems. This approach is a direct microbiome manipulation where inoculated bacterial consortia may serve to reduce the time required for the rhizosphere microbiome to achieve niche saturation and competitive exclusion of pathogens (Bakker et al., 2012). Further developing insight on our growth stage-related core bacteria and their potential benefits, microbial ecologists could design rationally optimized microbial consortia potentially with a high degree of effectiveness and persistence in the environment. Even if such inoculant design is based on the assessment of the close association with the host plant potentially in its target production area or region, unforeseen risks associated with bringing in inoculants on resident biodiversity and ecosystem functions (Hart et al., 2018) should be considered. While it is promising to see relatively high heritability estimate of broad microbial traits, the differential abundance and core bacterial analysis should expand toward identifying co-occurrence patterns, microbial hubs, evaluating the heritability of these taxa and linking them with plant genotype through genome-wide association studies. To conclude, this study has shown that in a realistic field setting plant genetics influences the canola microbiome, opening the promise of revolutionary ways to enhance canola productivity and sustainability.

## Data Availability Statement

The datasets generated for this study can be accessed from the NCBI Sequence Read Archive (https://www.ncbi.nlm.nih.gov/Traces/study/?acc=PRJNA575004) under accession number PRJNA575004.

## Author Contributions

SS and BH conceived and designed the experiments. SS, BH, ZT, EL, CN, MA, SV, IP, and JB performed the experiments. JB contributed in sequence library preparation and approved the manuscript. ZT, EL, TD, SM, SS, and ML designed bioinformatics pipelines for sequence data processing. ZT completed the final bioinformatics analysis. IP carried out the SNP analyses. SR created the genetic similarity matrix. ZT and EL set the objectives, analyzed the data, and wrote the manuscript. SS, BH, SV, IP, MA, CN, SM, ML, SR, and TD reviewed, commented, and approved the manuscript.

## Conflict of Interest

The authors declare that the research was conducted in the absence of any commercial or financial relationships that could be construed as a potential conflict of interest.
