# Supplementary material for: Core and Differentially Abundant Bacterial Taxa in the Rhizosphere of Field Grown Brassica napus Genotypes: Implications for Canola Breeding
Source: Front Microbiol. 2020 Jan 15;10:3007. doi: 10.3389/fmicb.2019.03007 (PMC6974584; doi:10.3389/fmicb.2019.03007)
Supplement: Supplementary file 1 [file Data_Sheet_1.PDF]

```
#Title:Core and Differentially Abundant Bacterial Taxa in the Rhizosphere of Field
Grown Brassica napus Genotypes:
#Implications for Canola Breeding
#Taye et al., 2019

## Differential abundance analysis
## Install edgeR and all other dependencies if you have not already

source("https://bioconductor.org/biocLite.R")
biocLite("edgeR")

## load all required packages
## load required ackage: edgeR, ggplot2
#library(phyloseq)
#library(edgeR)
#library(ggplot2)

##############################            #######
##############################
## load the phyloseq object created
## convert the abundace values to the nearest intigers. EdgeR accepts only
integers.
## we have spiked in A.fisheri during PCR amplification and latter used for
normalization
#to acount for variation in sequecnign depth.
## The normalized values are no longer integers. Hence, we rounded the abundance
value to nearest integer

canola2016_nearest_integer<-ceiling(otu_table(can_2016.norm.soil_zeor_fintered)) #
'can_2016.norm.soil_zeor_fintered' is our phyloseq object.

## merge the otu table with integer values with taxa table and meta data


can.physq.data.2016.nearest.intiger <-
merge_phyloseq(canola2016_nearest_integer,tax_table(can_2016.norm.soil_zeor_fintere
d),

sample_data(can_2016.norm.soil_zeor_fintered))

#save nearest intiger as RData# for later use as needed

#save(canola2016_nearest_integer,file='T:/_Docs/R_scripts/Annotated_r_scripts_and_w
orking_r_objects/canola2016_nearest_integer.RData')

## Aglomerate to a genus level
## Differencial abundace analysis will be done at genus level.The following
function will aggregrate taxa to Genus level

physeqGenus <- tax_glom(can.physq.data.2016.nearest.intiger, "Genus")
```

```
#save agglomerated physeqGenus object # for later use as needed

save(physeqGenus,file='D:/out_puts/phyloseq/with_green_genes_taxa/summary_tables/differential_abundance/physeqGenus_gg.RData')

##Check the phyloseq object (physeqGenus) you created: geneus level

physeqGenus ## will show summary of the phyloseq object

#phyloseq-class experiment-level object
#otu_table()   OTU Table:         [ 558 taxa and 477 samples ]
#sample_data() Sample Data:       [ 477 samples by 22 sample variables ]
#tax_table()   Taxonomy Table:    [ 558 taxa by 7 taxonomic ranks ]


#Get variables of interest from your phyloseq object
## we are interested to compare canola genotypes and hence "CanolaLine" is our variable of interest

canolaline = get_variable(physeqGenus, "CanolaLine")
head(canolaline)

##Now get your otutable from the phyloseq object. If it does not run add 1 to avoide log(0) error:over flow
## we have added 1

x = as(otu_table(physeqGenus), "matrix") + 1L
taxonomy = data.frame(as(tax_table(physeqGenus), "matrix"))

## Now you can turn into a DGEList

x = DGEList(counts=x, group=canolaline, genes=taxonomy, remove.zeros=TRUE)

## you can explore the DGEList created
x$samples

## Calculate normalization factro. Through our experimental design we have acounted for variation in sequencinng depth
# by normalizing the abundance values using the spikein. We tried different normalization methods implemented in edgeR
#and we did not find noticeable variation form plots (see plotBCV), finally we decided to aligning the upper quantiles
#of the count per million within the libraries.

x = calcNormFactors(x, method="upperquartile")

## you can explore the value of the normalization

x$samples
```

```
##Before proceeding to the differential abundance analysis lets see the sample
relations in multidimentional scaling


plotMDS(x, method="bcv", col=as.numeric(canolaline))

## Specify the design matrix: canola genotype is our trait of interest and as such
we have specified the design

design = model.matrix(~canolaline)

## save the desgn matrix for your reference

head(design)

write.csv(design,
'D:/out_puts/phyloseq/with_green_genes_taxa/summary_tables/differential_abundance/d
esign.csv')

## Disperssion estimate
#estimateDisp function runs all three estimates and is indicated as better rather
than running indiviudually.

library(statmod) # load statmod

x=estimateDisp(x,design, robust=TRUE) #robust=TRUE has been used to protect the
empirical Bayes estimates against the possibility of outlier taxa with
exceptionally large or small individual dispersions.

# visualize disperssion estimates using  BCV plot

plotBCV(x)

##Fit the negative binomial model with the specified design


fit <- glmQLFit(x, design)

##You can examine the fitted coeficients

head(fit$coefficients)

plotQLDisp(fit) #visualize the fitted mean-QL disperssion trend, squeezed QL
estimates


## Now proceed to determining differentially expressed genus. Here quasi-likelihood
F-tests is fitted.
#First a Genus wise glm is fitted and quasi-likelihood F-test comparing
```

```
#pairs of canola lines (NAM-0 VS NAM13 (COEF=2 indicates NAM-13 since it is in the 2nd column of the design matrix)) is condcuted.
#This will be done for all pair of comparisons, The reference genotype NAM-0 vs the remaining fiftteen genotypes.

############# NAM-0 Vs NAM-13###############

qlf <- glmQLFTest(fit, coef=2)
topTags(qlf)# to look at the toptags

##Now lets specify a false FDR treshold and take only those significant at this value. alpha 0.01

qlfnam13 = topTags(qlf, n=nrow(x), adjust.method="BH", sort.by="PValue")
qlfnam13 = qlfnam13@.Data[[1]]
alpha = 0.01
sigtab = qlfnam13[(qlfnam13$FDR < alpha), ]
sigtab = cbind(as(sigtab, "data.frame"),
as(tax_table(physeqGenus)[rownames(sigtab), ], "matrix"))
dim(sigtab) # prints the number of differentially abundant genera at FDR of 0.01
head(sigtab)# you can have a quick look at the head of the table with differentially significantlly abundant genera in NAM-13

##There is a separate script for extracting and better plotting edgeR results.Use this once for a quick overview

# Plot log-fold change against log-counts per million, with DE genus highlighted FOR NAM13.

detags <- rownames(sigtab)
plotSmear(qlf, de.tags=detags)
abline(h=c(-3, 3), col="blue")

## get the number of significanty more abundant and less abundant genus at adj pvalue= 0.01

nam13res=decideTestsDGE(qlf,adjust.method="BH", p.value=0.01)

summary(nam13res)

#Visualize the significnat DE GENUS IN NAM13. Use separate scrip for better plotting

sigtanam13 = subset(sigtab, !is.na(Genus))
# Phylum order
m = tapply(sigtanam13$logFC, sigtanam13$Phylum, function(y) max(y))
m = sort(m, TRUE)
sigtanam13$Phylum = factor(as.character(sigtanam13$Phylum), levels = names(m))
# Genus order
m = tapply(sigtanam13$logFC, sigtanam13$Genus, function(m) max(m))
```

```
m = sort(m, TRUE)
sigtanam13$Genus = factor(as.character(sigtanam13$Genus), levels = names(m))
ggplot(sigtanam13, aes(x = Genus, y = logFC, color = Phylum)) + geom_point(size=8)
+
  theme(axis.text.x = element_text(angle = -90, hjust = 0, vjust = 0.5)) +
  ggtitle("Log Fold Change of Significant Genus in NAM-13")


## save the significant DE geneus in NAME13 as csv in your folder/WD

write.csv(sigtab,
'D:/out_puts/phyloseq/with_green_genes_taxa/summary_tables/differential_abundance/N
AM_13_line_gg.csv')

##

############# NAM-0 Vs NAM-14###############

qlf1 <- glmQLFTest(fit, coef=3)
topTags(qlf1)

##Now lets specify a false FDR treshold and take only those significant at this
value. alpha 0.01

qlfnam14 = topTags(qlf1, n=nrow(x), adjust.method="BH", sort.by="PValue")
qlfnam14 = qlfnam14@.Data[[1]]
alpha = 0.01
sigtab1 = qlfnam14[(qlfnam14$FDR < alpha), ]
sigtab1 = cbind(as(sigtab1, "data.frame"),
as(tax_table(physeqGenus)[rownames(sigtab1), ], "matrix"))
dim(sigtab1)
head(sigtab1)#you can have a quick look at the head of the table with
differentially significantlly abundant genera in NAM-14

## Plot log-fold change against log-counts per million, with DE genus highlighted
FOR NAM14

detags1 <- rownames(sigtab1)
plotSmear(qlf1, de.tags=detags1)
abline(h=c(-3, 3), col="blue")

#number of up, down and not significant
nam14res=decideTestsDGE(qlf1,adjust.method="BH", p.value=0.01)
summary(nam14res)

## Visualize the significnat DE GENUS IN NAM14

sigtanam14 = subset(sigtab1, !is.na(Genus))
# Phylum order
j = tapply(sigtanam14$logFC, sigtanam14$Phylum, function(j) max(j))
```

```
j = sort(j, TRUE)
sigtanam14$Phylum = factor(as.character(sigtanam14$Phylum), levels = names(j))
# Genus order
j = tapply(sigtanam14$logFC, sigtanam14$Genus, function(j) max(j))
j = sort(j, TRUE)
sigtanam14$Genus = factor(as.character(sigtanam14$Genus), levels = names(j))
ggplot(sigtanam14, aes(x = Genus, y = logFC, color = Phylum)) + geom_point(size=8)
+
  theme(axis.text.x = element_text(angle = -90, hjust = 0, vjust = 0.5)) +
  ggtitle("Log Fold Change of Significant Genus in NAM-14")

## save the significant DE geneus in NAME14 as csv in your folder/WD

write.csv(sigtab1,
'D:/out_puts/phyloseq/with_green_genes_taxa/summary_tables/differential_abundance/N
AM_14_line_gg.csv')


############# NAM-0 Vs NAM-17###############

qlf2 <- glmQLFTest(fit, coef=4)
topTags(qlf2)

##Now lets specify a false FDR treshold and take only those significant at this
value. alpha 0.01
qlfnam17 = topTags(qlf2, n=nrow(x), adjust.method="BH", sort.by="PValue")
qlfnam17 = qlfnam17@.Data[[1]]
alpha = 0.01
sigtab2 = qlfnam17[(qlfnam17$FDR < alpha), ]
sigtab2 = cbind(as(sigtab2, "data.frame"),
as(tax_table(physeqGenus)[rownames(sigtab2), ], "matrix"))
dim(sigtab2)
head(sigtab2)

## Plot log-fold change against log-counts per million, with DE genus highlighted
FOR NAM17

detags2 <- rownames(sigtab2)
plotSmear(qlf2, de.tags=detags2)
abline(h=c(-1, 1), col="blue")

#number of up, down and not significant geneus

nam17res=decideTestsDGE(qlf2,adjust.method="BH", p.value=0.01)

summary(nam17res)

## Visualize the significnat DE GENUS IN NAM17
sigtanam17 = subset(sigtab2, !is.na(Genus))
# Phylum order
```

```
k = tapply(sigtanam17$logFC, sigtanam17$Phylum, function(k) max(k))
k = sort(k, TRUE)
sigtanam17$Phylum = factor(as.character(sigtanam17$Phylum), levels = names(k))
# Genus order
k = tapply(sigtanam17$logFC, sigtanam17$Genus, function(k) max(k))
k = sort(k, TRUE)
sigtanam17$Genus = factor(as.character(sigtanam17$Genus), levels = names(k))
ggplot(sigtanam17, aes(x = Genus, y = logFC, color = Phylum)) + geom_point(size=8)
+
  theme(axis.text.x = element_text(angle = -90, hjust = 0, vjust = 0.5)) +
  ggtitle("Log Fold Change of Significant Genus in NAM-17")

## save the significant DE geneus in NAM17 as csv in your folder/WD

write.csv(sigtab2,
'D:/out_puts/phyloseq/with_green_genes_taxa/summary_tables/differential_abundance/N
AM_17_line_gg.csv')


############# NAM-0 Vs NAM-23###############

qlf3 <- glmQLFTest(fit, coef=5)
topTags(qlf3)

#Here lets specify a FDR treshold and take only those significant at this value.
alpha 0.01

qlfnam23 = topTags(qlf3, n=nrow(x), adjust.method="BH", sort.by="PValue")
qlfnam23 = qlfnam23@.Data[[1]]
alpha = 0.01
sigtab3 = qlfnam23[(qlfnam23$FDR < alpha), ]
sigtab3 = cbind(as(sigtab3, "data.frame"),
as(tax_table(physeqGenus)[rownames(sigtab3), ], "matrix"))
dim(sigtab3)
head(sigtab3)

## Plot log-fold change against log-counts per million, with DE genus highlighted
FOR NAM23

detags3 <- rownames(sigtab3)
plotSmear(qlf3, de.tags=detags3)
abline(h=c(-1, 1), col="blue")

#number of up, down, not significnat genus

nam23res=decideTestsDGE(qlf3,adjust.method="BH", p.value=0.01)
summary(nam23res)

## Visualize the significnat DE GENUS IN NAM23
```

```
sigtanam23 = subset(sigtab3, !is.na(Genus))
# Phylum order
l = tapply(sigtanam23$logFC, sigtanam23$Phylum, function(l) max(l))
l = sort(l, TRUE)
sigtanam23$Phylum = factor(as.character(sigtanam23$Phylum), levels = names(l))
# Genus order
l = tapply(sigtanam23$logFC, sigtanam23$Genus, function(l) max(l))
l = sort(l, TRUE)
sigtanam23$Genus = factor(as.character(sigtanam23$Genus), levels = names(l))
ggplot(sigtanam23, aes(x = Genus, y = logFC, color = Phylum)) + geom_point(size=8)
+
  theme(axis.text.x = element_text(angle = -90, hjust = 0, vjust = 0.5)) +
  ggtitle("Log Fold Change of Significant Genus in NAM-23")

## save the significant DE geneus in NAME30 as csv in your folder/WD

write.csv(sigtab3,
'D:/out_puts/phyloseq/with_green_genes_taxa/summary_tables/differential_abundance/N
AM_23_line_gg.csv')

############# NAM-0 Vs NAM-30###############

qlf4 <- glmQLFTest(fit, coef = 6)
topTags(qlf4)

#Here lets specify a FDR treshold and take only those significant at this value.
alpha 0.01

qlfnam30 = topTags(qlf4, n=nrow(x), adjust.method="BH", sort.by="PValue")
qlfnam30 = qlfnam30@.Data[[1]]
alpha = 0.01
sigtab4 = qlfnam30[(qlfnam30$FDR < alpha), ]
sigtab4 = cbind(as(sigtab4, "data.frame"),
as(tax_table(physeqGenus)[rownames(sigtab4), ], "matrix"))
dim(sigtab4)
head(sigtab4)

## Plot log-fold change against log-counts per million, with DE genus highlighted
FOR NAM30

detags4 <- rownames(sigtab4)
plotSmear(qlf4, de.tags=detags4)
abline(h=c(-1, 1), col="blue")

#number of up, down, and not significant

nam30res=decideTestsDGE(qlf4,adjust.method="BH", p.value=0.01)
summary(nam30res)

## Visualize the significnat DE GENUS IN NAM30
```

```
sigtanam30 = subset(sigtab4, !is.na(Genus))
# Phylum order
b = tapply(sigtanam30$logFC, sigtanam30$Phylum, function(b) max(b))
b = sort(b, TRUE)
sigtanam30$Phylum = factor(as.character(sigtanam30$Phylum), levels = names(b))
# Genus order
b = tapply(sigtanam30$logFC, sigtanam30$Genus, function(b) max(b))
b = sort(b, TRUE)
sigtanam30$Genus = factor(as.character(sigtanam30$Genus), levels = names(b))
ggplot(sigtanam30, aes(x = Genus, y = logFC, color = Phylum)) + geom_point(size=8)
+
  theme(axis.text.x = element_text(angle = -90, hjust = 0, vjust = 0.5)) +
  ggtitle("Log Fold Change of Significant Genus in NAM-30")

## save the significant DE geneus in NAME32 as csv in your folder/WD
write.csv(sigtab4,
'D:/out_puts/phyloseq/with_green_genes_taxa/summary_tables/differential_abundance/N
AM_30_line_gg.csv')

############# NAM-0 Vs NAM-32###############

qlf5 <- glmQLFTest(fit, coef=7)
topTags(qlf5)

##Here lets specify a FDR treshold and take only those significant at this value.
alpha 0.01

qlfnam32 = topTags(qlf5, n=nrow(x), adjust.method="BH", sort.by="PValue")
qlfnam32 = qlfnam32@.Data[[1]]
alpha = 0.01
sigtab5 = qlfnam32[(qlfnam32$FDR < alpha), ]
sigtab5 = cbind(as(sigtab5, "data.frame"),
as(tax_table(physeqGenus)[rownames(sigtab5), ], "matrix"))
dim(sigtab5)
head(sigtab5)

## Plot log-fold change against log-counts per million, with DE genus highlighted
FOR NAM32

detags5 <- rownames(sigtab5)
plotSmear(qlf5, de.tags=detags5)
abline(h=c(-1, 1), col="blue")

#number of up, down and not significant geneus
nam32res=decideTestsDGE(qlf5,adjust.method="BH", p.value=0.01)
summary(nam32res)

## Visualize the significnat DE GENUS IN NAM32
```

```
sigtanam32 = subset(sigtab5, !is.na(Genus))
# Phylum order
c = tapply(sigtanam32$logFC, sigtanam32$Phylum, function(c) max(c))
c = sort(c, TRUE)
sigtanam32$Phylum = factor(as.character(sigtanam32$Phylum), levels = names(c))
# Genus order
c = tapply(sigtanam32$logFC, sigtanam32$Genus, function(c) max(c))
c = sort(c, TRUE)
sigtanam32$Genus = factor(as.character(sigtanam32$Genus), levels = names(c))
ggplot(sigtanam32, aes(x = Genus, y = logFC, color = Phylum)) + geom_point(size=8)
+
  theme(axis.text.x = element_text(angle = -90, hjust = 0, vjust = 0.5)) +
  ggtitle("Log Fold Change of Significant Genus in NAM-32")

## save the significant DE geneus in NAME32 as csv in your folder/WD

write.csv(sigtab5,
'D:/out_puts/phyloseq/with_green_genes_taxa/summary_tables/differential_abundance/N
AM_32_line_gg.csv')

############# NAM-0 Vs NAM-37###############

qlf6 <- glmQLFTest(fit, coef=8)
topTags(qlf6)

#Here lets specify a FDR treshold and take only those significant at this value.
alpha 0.01

qlfnam37 = topTags(qlf6, n=nrow(x), adjust.method="BH", sort.by="PValue")
qlfnam37 = qlfnam37@.Data[[1]]
alpha = 0.01
sigtab6 = qlfnam37[(qlfnam37$FDR < alpha), ]
sigtab6 = cbind(as(sigtab6, "data.frame"),
as(tax_table(physeqGenus)[rownames(sigtab6), ], "matrix"))
dim(sigtab6)
head(sigtab6)

## Plot log-fold change against log-counts per million, with DE genus highlighted
FOR NAM37

detags6 <- rownames(sigtab6)
plotSmear(qlf6, de.tags=detags6)
abline(h=c(-1, 1), col="blue")

#number of up, down and not significant genus

nam37res=decideTestsDGE(qlf6,adjust.method="BH", p.value=0.01)
summary(nam37res)

## Visualize the significnat DE GENUS IN NAM37
```

```
sigtanam37 = subset(sigtab6, !is.na(Genus))
# Phylum order
d = tapply(sigtanam37$logFC, sigtanam37$Phylum, function(d) max(d))
d = sort(d, TRUE)
sigtanam37$Phylum = factor(as.character(sigtanam37$Phylum), levels = names(d))
# Genus order
d = tapply(sigtanam37$logFC, sigtanam37$Genus, function(d) max(d))
d = sort(d, TRUE)
sigtanam37$Genus = factor(as.character(sigtanam37$Genus), levels = names(d))
ggplot(sigtanam37, aes(x = Genus, y = logFC, color = Phylum)) + geom_point(size=8)
+
  theme(axis.text.x = element_text(angle = -90, hjust = 0, vjust = 0.5)) +
  ggtitle("Log Fold Change of Significant Genus in NAM-37")

## save the significant DE geneus in NAM37 as csv in your folder/WD

write.csv(sigtab6,
'D:/out_puts/phyloseq/with_green_genes_taxa/summary_tables/differential_abundance/N
AM_37_line_gg.csv')

############# NAM-0 Vs NAM-43###############

qlf7 <- glmQLFTest(fit, coef=9)
topTags(qlf7)

##Here lets specify a FDR treshold and take only those significant at this value.
alpha 0.01

qlfnam43 = topTags(qlf7, n=nrow(x), adjust.method="BH", sort.by="PValue")
qlfnam43 = qlfnam43@.Data[[1]]
alpha = 0.01
sigtab7 = qlfnam43[(qlfnam43$FDR < alpha), ]
sigtab7 = cbind(as(sigtab7, "data.frame"),
as(tax_table(physeqGenus)[rownames(sigtab7), ], "matrix"))
dim(sigtab7)
head(sigtab7)

## Plot log-fold change against log-counts per million, with DE genus highlighted
FOR NAM43

detags7 <- rownames(sigtab7)
plotSmear(qlf7, de.tags=detags7)
abline(h=c(-1, 1), col="blue")

## number of up, down and not signifant taxa

nam43res=decideTestsDGE(qlf7,adjust.method="BH", p.value=0.01)
summary(nam43res)
```

```
## Visualize the significnat DE GENUS IN NAM43

sigtanam43 = subset(sigtab7, !is.na(Genus))
# Phylum order
f = tapply(sigtanam43$logFC, sigtanam43$Phylum, function(f) max(f))
f = sort(f, TRUE)
sigtanam43$Phylum = factor(as.character(sigtanam43$Phylum), levels = names(f))
# Genus order
f = tapply(sigtanam43$logFC, sigtanam43$Genus, function(f) max(f))
f = sort(f, TRUE)
sigtanam43$Genus = factor(as.character(sigtanam43$Genus), levels = names(f))
ggplot(sigtanam43, aes(x = Genus, y = logFC, color = Phylum)) + geom_point(size=8)
+
  theme(axis.text.x = element_text(angle = -90, hjust = 0, vjust = 0.5)) +
  ggtitle("Log Fold Change of Significant Genus in NAM-43")

## save the significant DE geneus in NAME43 as csv in your folder/WD

write.csv(sigtab7,
'D:/out_puts/phyloseq/with_green_genes_taxa/summary_tables/differential_abundance/N
AM_43_line_gg.csv')


############# NAM-0 Vs NAM-46###############

qlf8 <- glmQLFTest(fit, coef=10)
topTags(qlf8)

##Here lets specify a FDR treshold and take only those significant at this value.
alpha 0.01

qlfnam46 = topTags(qlf8, n=nrow(x), adjust.method="BH", sort.by="PValue")
qlfnam46 = qlfnam46@.Data[[1]]
alpha = 0.01
sigtab8 = qlfnam46[(qlfnam46$FDR < alpha), ]
sigtab8 = cbind(as(sigtab8, "data.frame"),
as(tax_table(physeqGenus)[rownames(sigtab8), ], "matrix"))
dim(sigtab8)
head(sigtab8)

## Plot log-fold change against log-counts per million, with DE genus highlighted
FOR NAM46

detags8 <- rownames(sigtab8)
plotSmear(qlf8, de.tags=detags8)
abline(h=c(-1, 1), col="blue")

# number of up, down and not significant genus

nam46res=decideTestsDGE(qlf8,adjust.method="BH", p.value=0.01)
```

```
summary(nam46res)

## Visualize the significnat DE GENUS IN NAM46

sigtanam46 = subset(sigtab8, !is.na(Genus))
# Phylum order
g = tapply(sigtanam46$logFC, sigtanam46$Phylum, function(g) max(g))
g = sort(g, TRUE)
sigtanam46$Phylum = factor(as.character(sigtanam46$Phylum), levels = names(g))
# Genus order
g = tapply(sigtanam46$logFC, sigtanam46$Genus, function(g) max(g))
g = sort(g, TRUE)
sigtanam46$Genus = factor(as.character(sigtanam46$Genus), levels = names(g))
ggplot(sigtanam46, aes(x = Genus, y = logFC, color = Phylum)) + geom_point(size=8)
+
  theme(axis.text.x = element_text(angle = -90, hjust = 0, vjust = 0.5)) +
  ggtitle("Log Fold Change of Significant Genus in NAM-46")

## save the significant DE geneus in NAME43 as csv in your folder/WD

write.csv(sigtab8,
'D:/out_puts/phyloseq/with_green_genes_taxa/summary_tables/differential_abundance/N
AM_46_line_gg.csv')

############# NAM-0 Vs NAM-48###############

qlf9 <- glmQLFTest(fit, coef=11)
topTags(qlf9)

##Here lets specify a FDR treshold and take only those significant at this value.
alpha 0.01

qlfnam48 = topTags(qlf9, n=nrow(x), adjust.method="BH", sort.by="PValue")
qlfnam48 = qlfnam48@.Data[[1]]
alpha = 0.01
sigtab9 = qlfnam48[(qlfnam48$FDR < alpha), ]
sigtab9 = cbind(as(sigtab9, "data.frame"),
as(tax_table(physeqGenus)[rownames(sigtab9), ], "matrix"))
dim(sigtab9)
head(sigtab9)

## Plot log-fold change against log-counts per million, with DE genus highlighted
FOR NAM48

detags9 <- rownames(sigtab9)
plotSmear(qlf9, de.tags=detags9)
abline(h=c(-1, 1), col="blue")

#number of up, down and not significant
```

```
nam48res=decideTestsDGE(qlf9,adjust.method="BH", p.value=0.01)
summary(nam48res)

## Visualize the significnat DE GENUS IN NAM48

sigtanam48 = subset(sigtab9, !is.na(Genus))
# Phylum order
h = tapply(sigtanam48$logFC, sigtanam48$Phylum, function(h) max(h))
h = sort(h, TRUE)
sigtanam48$Phylum = factor(as.character(sigtanam48$Phylum), levels = names(h))
# Genus order
h = tapply(sigtanam48$logFC, sigtanam48$Genus, function(h) max(h))
h = sort(h, TRUE)
sigtanam48$Genus = factor(as.character(sigtanam48$Genus), levels = names(h))
ggplot(sigtanam48, aes(x = Genus, y = logFC, color = Phylum)) + geom_point(size=8)
+
  theme(axis.text.x = element_text(angle = -90, hjust = 0, vjust = 0.5)) +
  ggtitle("Log Fold Change of Significant Genus in NAM-48")

## save the significant DE geneus in NAME5 as csv in your folder/WD

write.csv(sigtab9,
'D:/out_puts/phyloseq/with_green_genes_taxa/summary_tables/differential_abundance/N
AM_48_line_gg.csv')

############# NAM-0 Vs NAM-5###############

qlf10 <- glmQLFTest(fit, coef=12)
topTags(qlf10)

####Here lets specify a FDR treshold and take only those significant at this value.
alpha 0.01

qlfnam5 = topTags(qlf10, n=nrow(x), adjust.method="BH", sort.by="PValue")
qlfnam5 = qlfnam5@.Data[[1]]
alpha = 0.01
sigtab10 = qlfnam5[(qlfnam5$FDR < alpha), ]
sigtab10 = cbind(as(sigtab10, "data.frame"),
as(tax_table(physeqGenus)[rownames(sigtab10), ], "matrix"))
dim(sigtab10)
head(sigtab10)

## Plot log-fold change against log-counts per million, with DE genus highlighted
FOR NAM5

detags10 <- rownames(sigtab10)
plotSmear(qlf10, de.tags=detags10)
abline(h=c(-1, 1), col="blue")

#number of up, down and not significant genus
```

```
nam5res=decideTestsDGE(qlf10,adjust.method="BH", p.value=0.01)
summary(nam5res)

## Visualize the significnat DE GENUS IN NAM5

sigtanam5 = subset(sigtab10, !is.na(Genus))
# Phylum order
i = tapply(sigtanam5$logFC, sigtanam5$Phylum, function(i) max(i))
i = sort(i, TRUE)
sigtanam5$Phylum = factor(as.character(sigtanam5$Phylum), levels = names(i))
# Genus order
i = tapply(sigtanam5$logFC, sigtanam5$Genus, function(i) max(i))
i = sort(i, TRUE)
sigtanam5$Genus = factor(as.character(sigtanam5$Genus), levels = names(i))
ggplot(sigtanam5, aes(x = Genus, y = logFC, color = Phylum)) + geom_point(size=8) +

  theme(axis.text.x = element_text(angle = -90, hjust = 0, vjust = 0.5)) +
  ggtitle("Log Fold Change of Significant Genus in NAM-5")

## save the significant DE geneus in NAME5 as csv in your folder/WD

write.csv(sigtab10,
'D:/out_puts/phyloseq/with_green_genes_taxa/summary_tables/differential_abundance/N
AM_5_line_gg.csv')


############# NAM-0 Vs NAM-72###############

qlf11 <- glmQLFTest(fit, coef=13)
topTags(qlf11)

##Here lets specify a FDR treshold and take only those significant at this value.
alpha 0.01

qlfnam72 = topTags(qlf11, n=nrow(x), adjust.method="BH", sort.by="PValue")
qlfnam72 = qlfnam72@.Data[[1]]
alpha = 0.01
sigtab11 = qlfnam72[(qlfnam72$FDR < alpha), ]
sigtab11 = cbind(as(sigtab11, "data.frame"),
as(tax_table(physeqGenus)[rownames(sigtab11), ], "matrix"))
dim(sigtab11)
head(sigtab11)

## Plot log-fold change against log-counts per million, with DE genus highlighted
FOR NAM72

detags11 <- rownames(sigtab11)
plotSmear(qlf11, de.tags=detags11)
abline(h=c(-1, 1), col="blue")
```

```
#number of up, down and not significant genus

nam72res=decideTestsDGE(qlf11,adjust.method="BH", p.value=0.01)

summary(nam72res)

## Visualize the significnat DE GENUS IN NAM72

sigtanam72 = subset(sigtab11, !is.na(Genus))
# Phylum order
n = tapply(sigtanam72$logFC, sigtanam72$Phylum, function(n) max(n))
n = sort(n, TRUE)
sigtanam72$Phylum = factor(as.character(sigtanam72$Phylum), levels = names(n))
# Genus order
n = tapply(sigtanam72$logFC, sigtanam72$Genus, function(n) max(n))
n = sort(n, TRUE)
sigtanam72$Genus = factor(as.character(sigtanam72$Genus), levels = names(n))
ggplot(sigtanam72, aes(x = Genus, y = logFC, color = Phylum)) + geom_point(size=8)
+
  theme(axis.text.x = element_text(angle = -90, hjust = 0, vjust = 0.5)) +
  ggtitle("Log Fold Change of Significant Genus in NAM-72")

## save the significant DE geneus in NAME72 as csv in your folder/WD

write.csv(sigtab11,
'D:/out_puts/phyloseq/with_green_genes_taxa/summary_tables/differential_abundance/N
AM_72_line_gg.csv')

############# NAM-0 Vs NAM-76###############

qlf12 <- glmQLFTest(fit, coef=14)
topTags(qlf12)

##Here lets specify a FDR treshold and take only those significant at this value.
alpha 0.01

qlfnam76 = topTags(qlf12, n=nrow(x), adjust.method="BH", sort.by="PValue")
qlfnam76 = qlfnam76@.Data[[1]]
alpha = 0.01
sigtab12 = qlfnam76[(qlfnam76$FDR < alpha), ]
sigtab12 = cbind(as(sigtab12, "data.frame"),
as(tax_table(physeqGenus)[rownames(sigtab12), ], "matrix"))
dim(sigtab12)
head(sigtab12)

## Plot log-fold change against log-counts per million, with DE genus highlighted
FOR NAM76

detags12 <- rownames(sigtab12)
```

```
plotSmear(qlf12, de.tags=detags12)
abline(h=c(-1, 1), col="blue")

#number of up, down and not significant

nam76res=decideTestsDGE(qlf12,adjust.method="BH", p.value=0.01)
summary(nam76res)

### Visualize the significnat DE GENUS IN NAM76

sigtanam76 = subset(sigtab12, !is.na(Genus))
# Phylum order
p = tapply(sigtanam76$logFC, sigtanam76$Phylum, function(p) max(p))
p = sort(p, TRUE)
sigtanam76$Phylum = factor(as.character(sigtanam76$Phylum), levels = names(p))
# Genus order
p = tapply(sigtanam76$logFC, sigtanam76$Genus, function(p) max(p))
p = sort(p, TRUE)
sigtanam76$Genus = factor(as.character(sigtanam76$Genus), levels = names(p))
ggplot(sigtanam76, aes(x = Genus, y = logFC, color = Phylum)) + geom_point(size=8)
+
  theme(axis.text.x = element_text(angle = -90, hjust = 0, vjust = 0.5)) +
  ggtitle("Log Fold Change of Significant Genus in NAM-76")


# save the significant DE geneus in NAME76 as csv in your folder/WD

write.csv(sigtab12,
'D:/out_puts/phyloseq/with_green_genes_taxa/summary_tables/differential_abundance/N
AM_76_line_gg.csv')


############# NAM-0 Vs NAM-79###############

qlf13 <- glmQLFTest(fit, coef=15)
topTags(qlf13)

##Here lets specify a FDR treshold and take only those significant at this value.
alpha 0.01

qlfnam79 = topTags(qlf13, n=nrow(x), adjust.method="BH", sort.by="PValue")
qlfnam79 = qlfnam79@.Data[[1]]
alpha = 0.01
sigtab13 = qlfnam79[(qlfnam79$FDR < alpha), ]
sigtab13 = cbind(as(sigtab13, "data.frame"),
as(tax_table(physeqGenus)[rownames(sigtab13), ], "matrix"))
dim(sigtab13)
head(sigtab13)

## Plot log-fold change against log-counts per million, with DE genus highlighted
```

FOR NAM79

```
detags13 <- rownames(sigtab13)
plotSmear(qlf13, de.tags=detags13)
abline(h=c(-1, 1), col="blue")

# number of up, down and not significant

nam79res=decideTestsDGE(qlf13,adjust.method="BH", p.value=0.01)
summary(nam79res)

## Visualize the significnat DE GENUS IN NAM79

sigtanam79 = subset(sigtab13, !is.na(Genus))
# Phylum order
q = tapply(sigtanam79$logFC, sigtanam79$Phylum, function(q) max(q))
q = sort(q, TRUE)
sigtanam79$Phylum = factor(as.character(sigtanam79$Phylum), levels = names(q))
# Genus order
q = tapply(sigtanam79$logFC, sigtanam79$Genus, function(q) max(q))
q = sort(q, TRUE)
sigtanam79$Genus = factor(as.character(sigtanam79$Genus), levels = names(q))
ggplot(sigtanam79, aes(x = Genus, y = logFC, color = Phylum)) + geom_point(size=8)
+
  theme(axis.text.x = element_text(angle = -90, hjust = 0, vjust = 0.5)) +
  ggtitle("Log Fold Change of Significant Genus in NAM-79")

# save the significant DE geneus in NAME79 as csv in your folder/WD

write.csv(sigtab13,
'D:/out_puts/phyloseq/with_green_genes_taxa/summary_tables/differential_abundance/N
AM_79_line_gg.csv')


############# NAM-0 Vs YN04_C1213(NAM-94)###############

qlf14 <- glmQLFTest(fit, coef=16)
topTags(qlf14)

##Here lets specify a FDR treshold and take only those significant at this value.
alpha 0.01

qlfnamYN04 = topTags(qlf14, n=nrow(x), adjust.method="BH", sort.by="PValue")
qlfnamYN04 = qlfnamYN04@.Data[[1]]
alpha = 0.01
sigtab14 = qlfnamYN04[(qlfnamYN04$FDR < alpha), ]
sigtab14 = cbind(as(sigtab14, "data.frame"),
as(tax_table(physeqGenus)[rownames(sigtab14), ], "matrix"))
dim(sigtab14)
head(sigtab14)
```

```
## Plot log-fold change against log-counts per million, with DE genus highlighted
FOR YN04-C1213

detags14 <- rownames(sigtab14)
plotSmear(qlf14, de.tags=detags14)
abline(h=c(-1, 1), col="blue")

# number of up, down and not significant

namyno4res=decideTestsDGE(qlf14,adjust.method="BH", p.value=0.01)
summary(namyno4res)

## Visualize the significnat DE GENUS IN YN04-C1213

sigtanamYN04 = subset(sigtab14, !is.na(Genus))
# Phylum order
r = tapply(sigtanamYN04$logFC, sigtanamYN04$Phylum, function(r) max(r))
r = sort(r, TRUE)
sigtanamYN04$Phylum = factor(as.character(sigtanamYN04$Phylum), levels = names(r))
# Genus order
r = tapply(sigtanamYN04$logFC, sigtanamYN04$Genus, function(r) max(r))
r = sort(r, TRUE)
sigtanamYN04$Genus = factor(as.character(sigtanamYN04$Genus), levels = names(r))
ggplot(sigtanamYN04, aes(x = Genus, y = logFC, color = Phylum)) +
geom_point(size=8) +
  theme(axis.text.x = element_text(angle = -90, hjust = 0, vjust = 0.5)) +
  ggtitle("Log Fold Change of Significant Genus in NAM-YN04")


# save the significant DE geneus in YN04-C1213 (NAM-94) as csv in your folder/WD

write.csv(sigtab14,
'D:/out_puts/phyloseq/with_green_genes_taxa/summary_tables/differential_abundance/Y
N04-C1213_line_gg.csv')

## save as image

save.image(file="T:/_Docs/R_scripts/Annotated_r_scripts_and_working_r_objects/canol
a.2016.differential.abundance.edger_2018finalpaper.RData")
```
