# Supplementary material for: Core and Differentially Abundant Bacterial Taxa in the Rhizosphere of Field Grown Brassica napus Genotypes: Implications for Canola Breeding
Source: Front Microbiol. 2020 Jan 15;10:3007. doi: 10.3389/fmicb.2019.03007 (PMC6974584; doi:10.3389/fmicb.2019.03007)
Supplement: Supplementary file 2 [file Data_Sheet_2.PDF]

```
#Title:Core and Differentially Abundant Bacterial Taxa in the Rhizosphere of Field Grown Brassica napus Genotypes:
#Implications for Canola Breeding
#Taye et al., 2019

# Core microbiome analysis
library(microbiome) ## Install microbiome R package and all dependencies if you have not installed
library(knitr)

# load the 2016 phyloseq object and convert absolute counts to compositional (relative) abundance

phyloseq.object.corrected.line.names.2016

pysqcdata.compositional <- transform(phyloseq.object.corrected.line.names.2016, "compositional")

## Core Microbiome with detection threshold of 0.01/100 and a series of prevalence tresholds ranging from 50 - 100/100

#### Core taxa at 50% prevalnce treshold

pysqcdata.core1 <- core(pysqcdata.compositional,
                    detection = .01/100, prevalence = 50/100)
pysqcdata.core1 ## this is a phyloseq object with core taxa identified with the specified tresholds

## Names of the core taxa based on the above specified detection and prevalence treshold

core.taxa <- taxa(pysqcdata.core1)
class(core.taxa)
# get the taxonomy data
tax.mat <- tax_table(pysqcdata.core1)
tax.df <- as.data.frame(tax.mat)
# add the OTus to last column
tax.df$OTU <- rownames(tax.df)

# select taxonomy of only
# those OTUs that are core memebers based on the thresholds that were used.
core.taxa.class1 <- dplyr::filter(tax.df, rownames(tax.df) %in% core.taxa)
knitr::kable(core.taxa.class1)

#Save csv of core microbiome at 50 prevalence treshold
write.csv(core.taxa.class1,
'D:/out_puts/phyloseq/with_green_genes_taxa/summary_tables/core_taxa_tables/core.taxa.50.gg.csv')

### Core taxa at 55% prevalnce treshold
```

```
pysqcdata.core2 <- core(pysqcdata.compositional,
                    detection = .01/100, prevalence = 55/100)
pysqcdata.core2

## Names of the core taxa based on the above specified detection and prevalence
treshold
core.taxa <- taxa(pysqcdata.core2)
class(core.taxa)
# get the taxonomy data
tax.mat <- tax_table(pysqcdata.core2)
tax.df <- as.data.frame(tax.mat)

# add the OTus to last column
tax.df$OTU <- rownames(tax.df)

# select taxonomy of only
# those OTUs that are core memebers based on the thresholds that were used.
core.taxa.class2 <- dplyr::filter(tax.df, rownames(tax.df) %in% core.taxa)
knitr::kable(core.taxa.class2)

write.csv(core.taxa.class2,
'D:/out_puts/phyloseq/with_green_genes_taxa/summary_tables/core_taxa_tables/core.ta
xa.55.gg.csv')

### Core taxa at 60% prevalnce treshold

pysqcdata.core3 <- core(pysqcdata.compositional,
                    detection = .01/100, prevalence = 60/100)
pysqcdata.core3

# Names of the core taxa based on the above specified detection and prevalence
treshold

core.taxa <- taxa(pysqcdata.core3)
class(core.taxa)
# get the taxonomy data
tax.mat <- tax_table(pysqcdata.core3)
tax.df <- as.data.frame(tax.mat)

# add the OTus to last column
tax.df$OTU <- rownames(tax.df)

# select taxonomy of only
# those OTUs that are core memebers based on the thresholds that were used.
core.taxa.class3 <- dplyr::filter(tax.df, rownames(tax.df) %in% core.taxa)
knitr::kable(core.taxa.class3)

write.csv(core.taxa.class3,
'D:/out_puts/phyloseq/with_green_genes_taxa/summary_tables/core_taxa_tables/core.ta
```

```
xa.60.gg.csv')

### Core taxa at 65% prevalnce treshold

pysqcdata.core4 <- core(pysqcdata.compositional,
                    detection = .01/100, prevalence = 65/100)
pysqcdata.core4

core.taxa <- taxa(pysqcdata.core4)
class(core.taxa)
# get the taxonomy data
tax.mat <- tax_table(pysqcdata.core4)
tax.df <- as.data.frame(tax.mat)

# add the OTus to last column
tax.df$OTU <- rownames(tax.df)

# select taxonomy of only
# those OTUs that are core memebers based on the thresholds that were used.
core.taxa.class4 <- dplyr::filter(tax.df, rownames(tax.df) %in% core.taxa)
knitr::kable(core.taxa.class4)

write.csv(core.taxa.class4,
'D:/out_puts/phyloseq/with_green_genes_taxa/summary_tables/core_taxa_tables/core.ta
xa.65.gg.csv')

### Core taxa at 70% prevalnce treshold

pysqcdata.core5 <- core(pysqcdata.compositional,
                    detection = .01/100, prevalence = 70/100)
pysqcdata.core5

# Names of the core taxa based on the above specified detection and prevalence
treshold
core.taxa <- taxa(pysqcdata.core5)
class(core.taxa)
# get the taxonomy data
tax.mat <- tax_table(pysqcdata.core5)
tax.df <- as.data.frame(tax.mat)

# add the OTus to last column
tax.df$OTU <- rownames(tax.df)

# select taxonomy of only
# those OTUs that are core memebers based on the thresholds that were used.
core.taxa.class5 <- dplyr::filter(tax.df, rownames(tax.df) %in% core.taxa)
knitr::kable(core.taxa.class5)

write.csv(core.taxa.class5,
'D:/out_puts/phyloseq/with_green_genes_taxa/summary_tables/core_taxa_tables/core.ta
```

```
xa.70.gg.csv')

### Core taxa at 75% prevalnce treshold

pysqcdata.core6 <- core(pysqcdata.compositional,
                    detection = .01/100, prevalence = 75/100)
pysqcdata.core6

# Names of the core taxa based on the above specified detection and prevalence
treshold

core.taxa <- taxa(pysqcdata.core6)
class(core.taxa)
# get the taxonomy data
tax.mat <- tax_table(pysqcdata.core6)
tax.df <- as.data.frame(tax.mat)

# add the OTus to last column
tax.df$OTU <- rownames(tax.df)

# select taxonomy of only
# those OTUs that are core memebers based on the thresholds that were used.
core.taxa.class6 <- dplyr::filter(tax.df, rownames(tax.df) %in% core.taxa)
knitr::kable(core.taxa.class6)

write.csv(core.taxa.class6,
'D:/out_puts/phyloseq/with_green_genes_taxa/summary_tables/core_taxa_tables/core.ta
xa.75.gg.csv')

### Core taxa at 80% prevalnce treshold

pysqcdata.core7 <- core(pysqcdata.compositional,
                    detection = .01/100, prevalence = 80/100)
pysqcdata.core7

# Names of the core taxa based on the above specified detection and prevalence
treshold
core.taxa <- taxa(pysqcdata.core7)
class(core.taxa)
# get the taxonomy data
tax.mat <- tax_table(pysqcdata.core7)
tax.df <- as.data.frame(tax.mat)

# add the OTus to last column
tax.df$OTU <- rownames(tax.df)

# select taxonomy of only
# those OTUs that are core memebers based on the thresholds that was used.
core.taxa.class7 <- dplyr::filter(tax.df, rownames(tax.df) %in% core.taxa)
knitr::kable(core.taxa.class7)
```

```
write.csv(core.taxa.class7,
'D:/out_puts/phyloseq/with_green_genes_taxa/summary_tables/core_taxa_tables/core.ta
xa.80.gg.csv')

### Core taxa at 85% prevalnce treshold

pysqcdata.core8 <- core(pysqcdata.compositional,
                    detection = .01/100, prevalence = 85/100)
pysqcdata.core8

# Names of the core taxa based on the above specified detection and prevalence
treshold

core.taxa <- taxa(pysqcdata.core8)
class(core.taxa)
# get the taxonomy data
tax.mat <- tax_table(pysqcdata.core8)
tax.df <- as.data.frame(tax.mat)

# add the OTus to last column
tax.df$OTU <- rownames(tax.df)

# select taxonomy of only
# those OTUs that are core memebers based on the thresholds that were used.
core.taxa.class8 <- dplyr::filter(tax.df, rownames(tax.df) %in% core.taxa)
knitr::kable(core.taxa.class8)

write.csv(core.taxa.class8,
'D:/out_puts/phyloseq/with_green_genes_taxa/summary_tables/core_taxa_tables/core.ta
xa.85.gg.csv')

### Core taxa at 90% prevalnce treshold

pysqcdata.core9 <- core(pysqcdata.compositional,
                    detection = .01/100, prevalence = 90/100)
pysqcdata.core9

# Names of the core taxa based on the above specified detection and prevalence
treshold

core.taxa <- taxa(pysqcdata.core9)
class(core.taxa)
# get the taxonomy data
tax.mat <- tax_table(pysqcdata.core9)
tax.df <- as.data.frame(tax.mat)

# add the OTus to last column
tax.df$OTU <- rownames(tax.df)
```

```
# select taxonomy of only
# those OTUs that are core memebers based on the thresholds that were used.
core.taxa.class9 <- dplyr::filter(tax.df, rownames(tax.df) %in% core.taxa)
knitr::kable(core.taxa.class9)

write.csv(core.taxa.class9,
'D:/out_puts/phyloseq/with_green_genes_taxa/summary_tables/core_taxa_tables/core.ta
xa.90.gg.csv')

### Core taxa at 95% prevalnce treshold

pysqcdata.core10 <- core(pysqcdata.compositional,
                     detection = .01/100, prevalence = 96/100)
pysqcdata.core10

# Names of the core taxa based on the above specified detection and prevalence
treshold

core.taxa <- taxa(pysqcdata.core10)
class(core.taxa)
# get the taxonomy data
tax.mat <- tax_table(pysqcdata.core10)
tax.df <- as.data.frame(tax.mat)

# add the OTus to last column
tax.df$OTU <- rownames(tax.df)

# select taxonomy of only
# those OTUs that are core memebers based on the thresholds that were used.
core.taxa.class10 <- dplyr::filter(tax.df, rownames(tax.df) %in% core.taxa)
knitr::kable(core.taxa.class10)

write.csv(core.taxa.class10,
'D:/out_puts/phyloseq/with_green_genes_taxa/summary_tables/core_taxa_tables/core.ta
xa.96.gg.csv')

#There were no core taxa at 100% prevalence treshold

#####################  //#######################################

## 2017 three site core microbiome analysis

### Site: LL

# Load your phyloseq object

myphylo_zero_sum_filtered_LL_17.RData

library(microbiome)
```

```
# convert absolute counts to compositional (relative) abundances
pysqcdata.compositional <-
transform(myphylo_zero_sum_filtered_LL_17"compositional")

### Core taxa at 75% prevalnce treshold

pysqcdata.core6 <- core(pysqcdata.compositional,
                    detection = .01/100, prevalence = 75/100)
pysqcdata.core6

# Names of the core taxa based on the above specified detection and prevalence
treshold

core.taxa <- taxa(pysqcdata.core6)
class(core.taxa)
# get the taxonomy data
tax.mat <- tax_table(pysqcdata.core6)
tax.df <- as.data.frame(tax.mat)

# add the OTus to last column
tax.df$OTU <- rownames(tax.df)

# select taxonomy of only
# those OTUs that are core memebers based on the thresholds that were used.
core.taxa.class6 <- dplyr::filter(tax.df, rownames(tax.df) %in% core.taxa)
knitr::kable(core.taxa.class6)

write.csv(core.taxa.class6,
'D:/out_puts/2017_canola/LL_qqime2_outputs/tables/core_taxa_tables/core.taxa.75.LL_
17.csv'

### Site: MF

# Load your phyloseq object

myphylo_zero_sum_filtered_MF_17.RData

# convert absolute counts to compositional (relative) abundances
pysqcdata.compositional <- transform(myphylo_zero_sum_filtered_MF_17,
"compositional")

### Core taxa at 75% prevalnce treshold

pysqcdata.core6 <- core(pysqcdata.compositional,
                    detection = .01/100, prevalence = 75/100)
pysqcdata.core6

# Names of the core taxa based on the above specified detection and prevalence
treshold
```

```
core.taxa <- taxa(pysqcdata.core6)
class(core.taxa)
# get the taxonomy data
tax.mat <- tax_table(pysqcdata.core6)
tax.df <- as.data.frame(tax.mat)

# add the OTus to last column
tax.df$OTU <- rownames(tax.df)

# select taxonomy of only
# those OTUs that are core memebers based on the thresholds that were used.
core.taxa.class6 <- dplyr::filter(tax.df, rownames(tax.df) %in% core.taxa)
knitr::kable(core.taxa.class6)

write.csv(core.taxa.class6,
'D:/out_puts/2017_canola/MF_qqime2_outputs/tables/core_taxa_tables/core.taxa.75.MF_
17.csv')


### Site: SC

# Load your phyloseq object

myphylo_zero_sum_filtered_SC_17.RData

# convert absolute counts to compositional (relative) abundances
pysqcdata.compositional <- transform(myphylo_zero_sum_filtered_SC_17,
"compositional")

### Core taxa at 75% prevalnce treshold

pysqcdata.core6 <- core(pysqcdata.compositional,
                    detection = .01/100, prevalence = 75/100)
pysqcdata.core6

# Names of the core taxa based on the above specified detection and prevalence
treshold

core.taxa <- taxa(pysqcdata.core6)
class(core.taxa)
# get the taxonomy data
tax.mat <- tax_table(pysqcdata.core6)
tax.df <- as.data.frame(tax.mat)

# add the OTus to last column
tax.df$OTU <- rownames(tax.df)

# select taxonomy of only
# those OTUs that are core memebers based on the thresholds that were used.
core.taxa.class6 <- dplyr::filter(tax.df, rownames(tax.df) %in% core.taxa)
```

```
knitr::kable(core.taxa.class6)

write.csv(core.taxa.class6,
'D:/out_puts/2017_canola/SC_qqime2_outputs/tables/core_taxa_tables/core.taxa.75.SC_
17.csv')

#########################################
//######################################################################
```
