# Supplementary material for: Core and Differentially Abundant Bacterial Taxa in the Rhizosphere of Field Grown Brassica napus Genotypes: Implications for Canola Breeding
Source: Front Microbiol. 2020 Jan 15;10:3007. doi: 10.3389/fmicb.2019.03007 (PMC6974584; doi:10.3389/fmicb.2019.03007)
Supplement: Supplementary file 3 [file Data_Sheet_3.PDF]

```
#Title:Core and Differentially Abundant Bacterial Taxa in the Rhizosphere of Field
Grown Brassica napus Genotypes:
#Implications for Canola Breeding
#Taye et al., 2019

# Heritability analysis

## Import the phyloseq object and EdgeR normalize
phyloseq.object.corrected.line.names.2016 # this is my phyloseq object

phyloseq.object.corrected.line.names.2016.edgernorm =
normalise_data(phyloseq.object.corrected.line.names.2016, norm.method =
"edgernorm")
phyloseq.object.corrected.line.names.2016.edgernorm

# Calculate all alpha diversity measures: use phyloseq and microbiome R packages
library(microbiome)
library(knitr)
library(dplyr)
library(ggpubr)

Alpha.can.2016 <- alpha(phyloseq.object.corrected.line.names.2016.edgernorm, index
= "all") ## alpha diversity based on whole data

kable(head(Alpha.can.2016))

# get metadata from the phyloseq object and add the calculated diversity measures
can.meta <- meta(phyloseq.object.corrected.line.names.2016.edgernorm)

kable(head(can.meta))

#observed|    chao1| diversity_inverse_simpson| diversity_gini_simpson|
diversity_shannon| diversity_fisher|
 # diversity_coverage| evenness_camargo| evenness_pielou| evenness_simpson|
evenness_evar| evenness_bulla| dominance_dbp|
  #dominance_dmn| dominance_absolute| dominance_relative| dominance_simpson|
dominance_core_abundance| dominance_gini|
#  rarity_log_modulo_skewness| rarity_low_abundance| rarity_noncore_abundance|
rarity_rare_abundance|

### Diversity_shannon, diversity_gini_simpson, diversity_inverse_simpson,
evenness_pielou and eveness_simpson
#were considered for heritability analysis. You can test the other diversity
measures if interested

can.meta$observed <- Alpha.can.2016$observed
can.meta$chao1 <- Alpha.can.2016$chao1
can.meta$diversity_inverse_simpson <- Alpha.can.2016$diversity_inverse_simpson
can.meta$diversity_gini_simpson <- Alpha.can.2016$diversity_gini_simpson
can.meta$diversity_shannon <- Alpha.can.2016$diversity_shannon
```

```
can.meta$diversity_fisher <- Alpha.can.2016$diversity_fisher
can.meta$diversity_coverage <- Alpha.can.2016$diversity_coverage
can.meta$evenness_camargo <- Alpha.can.2016$evenness_camargo
can.meta$evenness_pielou <- Alpha.can.2016$evenness_pielou
can.meta$evenness_simpson <- Alpha.can.2016$evenness_simpson
can.meta$evenness_evar <- Alpha.can.2016$evenness_evar
can.meta$evenness_bulla <- Alpha.can.2016$evenness_bulla
can.meta$dominance_dbp <- Alpha.can.2016$dominance_dbp
can.meta$dominance_dmn <- Alpha.can.2016$dominance_dmn
can.meta$dominance_absolute <- Alpha.can.2016$dominance_absolute
can.meta$dominance_relative <- Alpha.can.2016$dominance_relative
can.meta$dominance_simpson <- Alpha.can.2016$dominance_simpson
can.meta$dominance_core_abundance <- Alpha.can.2016$dominance_core_abundance
can.meta$dominance_gini <- Alpha.can.2016$dominance_gini
can.meta$rarity_log_modulo_skewness <- Alpha.can.2016$rarity_log_modulo_skewness
can.meta$rarity_low_abundance <- Alpha.can.2016$rarity_low_abundance
can.meta$rarity_noncore_abundance <- Alpha.can.2016$rarity_noncore_abundance
can.meta$rarity_rare_abundance <- Alpha.can.2016$rarity_rare_abundance

####
write.csv(can.meta,
'D:/out_puts/phyloseq/with_green_genes_taxa/microbiomeseq_analyssi/all_alpha_divers
ity_measures_microbiomer.csv')
Plot=as.factor(can.meta$Plot)
#### use the diversity measures to calculate broad-sense heritability
### variance components were extracted from each fitted models from which table 5
in manuscript is prepared
library(lme4)
#m1 = lmer(log(chao1)~(1|CanolaLine) + (1|CanolaLine:Weeks),
data=all_alpha_diversity_measures_microbiomer, REML = TRUE)
#summary(m1) #
#h1= (0.000001146)/((0.000001146) +(( 0.000016432)/10)+((0.000435987)/30))

m2 = lmer(diversity_inverse_simpson~(1|CanolaLine) + (1|CanolaLine:Weeks),
data=all_alpha_diversity_measures_microbiomer, REML = TRUE)
summary(m2)

h2= (46.79 )/((46.79 )+((204.49)/10)+((3688.61)/30))

m3 = lmer(diversity_gini_simpson~(1|CanolaLine) + (1|CanolaLine:Weeks),
data=all_alpha_diversity_measures_microbiomer, REML = TRUE)
summary(m3)
h3= (0.00002678)/((0.00002678) +((0.00002116)/10)+((0.00132184/30)))


m4 = lmer(diversity_shannon~(1|CanolaLine) + (1|CanolaLine:Weeks),
data=all_alpha_diversity_measures_microbiomer, REML = TRUE)
summary(m4)
h4= (0.003671)/((0.003671) +((0.019060)/10)+ ((0.542428)/30))
```

```
m5 = lmer(evenness_pielou~(1|CanolaLine) + (1|CanolaLine:Weeks),
data=all_alpha_diversity_measures_microbiomer, REML = TRUE)
summary(m5)
h5= ( 0.0000412)/( (0.0000412) +((0.0002139)/10) +((0.0060884)/30))


m6 = lmer(evenness_simpson~(1|CanolaLine) + (1|CanolaLine:Weeks),
data=all_alpha_diversity_measures_microbiomer, REML = TRUE)
summary(m6)
h6= (0.0000002963)/((0.0000002963) +((0.0000012948)/10) + ((0.0000233561)/30))

#m7 = lmer(dominance_dbp~(1|CanolaLine) + (1|CanolaLine:Weeks),
data=all_alpha_diversity_measures_microbiomer, REML = TRUE)
#summary(m7)
#h7= (0.0001648)/((0.0001648) +((0.0004511)/10) + ((0.0057704)/30))

#m8 = lmer(dominance_dmn~(1|CanolaLine) + (1|CanolaLine:Weeks),
data=all_alpha_diversity_measures_microbiomer, REML = TRUE)
#summary(m8)
#h8= (0.0002727)/((0.0002727) +(( 0.0007020)/10) + ((0.0105888)/30))

#m9 = lmer(dominance_relative~(1|CanolaLine) + (1|CanolaLine:Weeks),
data=all_alpha_diversity_measures_microbiomer, REML = TRUE)
#summary(m9)
#h9= (0.0001648)/((0.0001648) +(( 0.0004511)/10) + ((0.0057704)/30))

#m10 = lmer(dominance_simpson~(1|CanolaLine) + (1|CanolaLine:Weeks),
data=all_alpha_diversity_measures_microbiomer, REML = TRUE)
#summary(m10)
#h10= (0.00002678)/((0.00002678) +(( 0.00002116)/10) + ((0.00132184)/30))

#m11 = lmer(rarity_low_abundance~(1|CanolaLine) + (1|CanolaLine:Weeks),
data=all_alpha_diversity_measures_microbiomer, REML = TRUE)
#summary(m11)
#h11= (0.00002116 +0.00002678)/(0.00002116 +0.00002678 +0.0001648+0.00132184)

##################     //          ########################################

#### subset data to only flowering - calculate alpha diversity measures and
estimate heritability
can.Flowering.2016 = subset_samples(phyloseq.object.corrected.line.names.2016,
Week=="4"|Week=="5"|Week=="6"|Week=="7") ## subset to weeks 4,5,6,7:Flowering stage

can.Flowering.2016.edgernorm = normalise_data(can.Flowering.2016, norm.method =
"edgernorm")## normalize the data using edgeR normalization
Alpha.can.2016.Flowering <- alpha(can.Flowering.2016.edgernorm, index = "all")

# get metadata from the phyloseq object and add the calculated diversity measures
can.meta <- meta(can.Flowering.2016.edgernorm)
```

```
kable(head(can.meta))

#
can.meta$observed <- Alpha.can.2016.Flowering$observed
can.meta$chao1 <- Alpha.can.2016.Flowering$chao1
can.meta$diversity_inverse_simpson <-
Alpha.can.2016.Flowering$diversity_inverse_simpson
can.meta$diversity_gini_simpson <- Alpha.can.2016.Flowering$diversity_gini_simpson
can.meta$diversity_shannon <- Alpha.can.2016.Flowering$diversity_shannon
can.meta$diversity_fisher <- Alpha.can.2016.Flowering$diversity_fisher
can.meta$diversity_coverage <- Alpha.can.2016.Flowering$diversity_coverage
can.meta$evenness_camargo <- Alpha.can.2016.Flowering$evenness_camargo
can.meta$evenness_pielou <- Alpha.can.2016.Flowering$evenness_pielou
can.meta$evenness_simpson <- Alpha.can.2016.Flowering$evenness_simpson
can.meta$evenness_evar <- Alpha.can.2016.Flowering$evenness_evar
can.meta$evenness_bulla <- Alpha.can.2016.Flowering$evenness_bulla
can.meta$dominance_dbp <- Alpha.can.2016.Flowering$dominance_dbp
can.meta$dominance_dmn <- Alpha.can.2016.Flowering$dominance_dmn
can.meta$dominance_absolute <- Alpha.can.2016.Flowering$dominance_absolute
can.meta$dominance_relative <- Alpha.can.2016.Flowering$dominance_relative
can.meta$dominance_simpson <- Alpha.can.2016.Flowering$dominance_simpson
can.meta$dominance_core_abundance <-
Alpha.can.2016.Flowering$dominance_core_abundance
can.meta$dominance_gini <- Alpha.can.2016.Flowering$dominance_gini
can.meta$rarity_log_modulo_skewness <-
Alpha.can.2016.Flowering$rarity_log_modulo_skewness
can.meta$rarity_low_abundance <- Alpha.can.2016.Flowering$rarity_low_abundance
can.meta$rarity_noncore_abundance <-
Alpha.can.2016.Flowering$rarity_noncore_abundance
can.meta$rarity_rare_abundance <- Alpha.can.2016.Flowering$rarity_rare_abundance

####
write.csv(can.meta,
'D:/out_puts/phyloseq/with_green_genes_taxa/microbiomeseq_analyssi/all_alpha_divers
ity_measures_flowering_microbiomer.csv')

#
#### use the diversity measures to calculate broad-sense heritability at flowering
library(lme4)
#m11 = lmer(chao1~(1|CanolaLine),
data=all_alpha_diversity_measures_flowering_microbiomer, REML = TRUE)
#summary(m11) #model did not converge
#h11 = (123775943273)/(123775943273 + 2483337164217)

m12 = lmer(diversity_inverse_simpson~(1|CanolaLine),
data=all_alpha_diversity_measures_flowering_microbiomer, REML = TRUE)
summary(m12)

h12= (403.1)/((403.1) + ((3366.9)/12))
```

```
m13 = lmer(diversity_gini_simpson~(1|CanolaLine),
data=all_alpha_diversity_measures_flowering_microbiomer, REML = TRUE)
summary(m13)
h13= (0.00005502)/((0.00005502) + ((0.00114562)/12))


m14 = lmer(diversity_shannon~(1|CanolaLine),
data=all_alpha_diversity_measures_flowering_microbiomer, REML = TRUE)
summary(m14)
h14= (0.0489)/((0.0489) +((0.5645)/12))

m15 = lmer(evenness_pielou~(1|CanolaLine),
data=all_alpha_diversity_measures_flowering_microbiomer, REML = TRUE)
summary(m15)
h15= (0.0005489)/((0.0005489) + ((0.0063357)/12))

m16 = lmer(evenness_simpson~(1|CanolaLine),
data=all_alpha_diversity_measures_flowering_microbiomer, REML = TRUE)
summary(m16)
h16= (0.000002552)/((0.000002552) + ((0.000021319)/12))

#m17 = lmer(dominance_dbp~(1|CanolaLine),
data=all_alpha_diversity_measures_flowering_microbiomer, REML = TRUE)
#summary(m17)
#h17= (0.0003317)/((0.0003317) + ((0.0063080)/12))

#m18 = lmer(dominance_dmn~(1|CanolaLine),
data=all_alpha_diversity_measures_flowering_microbiomer, REML = TRUE)
#summary(m18)
#h18= (0.0008469)/((0.0008469) + ((0.0111165)/12))

#m19 = lmer(dominance_relative~(1|CanolaLine),
data=all_alpha_diversity_measures_flowering_microbiomer, REML = TRUE)
#summary(m19)
#h19= (0.0003317)/((0.0003317) + ((0.0063080)/12))

#m20 = lmer(dominance_simpson~(1|CanolaLine),
data=all_alpha_diversity_measures_flowering_microbiomer, REML = TRUE)
#summary(m20)
#h20= (0.00005502)/((0.00005502) + ((0.00114562)/12))

#m21 = lmer(rarity_low_abundance~(1|CanolaLine),
data=all_alpha_diversity_measures_flowering_microbiomer, REML = TRUE)
#summary(m21)
#h21= (0.0002578)/((0.0002578) + ((0.0097715)/12))

############################################
//#####################################################

##PERMANOVA, correlation between bray-curtis and genotype genetic distance.
```

```
phyloseq.object.corrected.line.names.2016 ## 2016 canola rhizosphere dataset

## Vegetative stage
can.vegetative.2016 = subset_samples(phyloseq.object.corrected.line.names.2016,
Week=="1"|Week=="2"|Week=="3") ## subset to weeks 1,2,3:vegetative stage

can.vegetative.2016.edgernorm = normalise_data(can.vegetative.2016, norm.method =
"edgernorm")## normalize the data using edgeR normalization

###PERMANOVA on the normalized data using adonis: using phyloseq #
set.seed(100)
can_bray.2016.veg <-phyloseq::distance(can.vegetative.2016.edgernorm,
                                   method = "bray")# calculate bray curtis distance
matrix
sampledf.vg = data.frame(sample_data(can.vegetative.2016.edgernorm))# make a data
frame from the sample_data of the phyloseq object
adonis(can_bray.2016.veg~Canola.Lines, data = sampledf.vg) # perform permanova test
using adonis function in Vegan ## is not significant- accepting that genotypes have
the same centroid
beta = betadisper(can_bray.2016.veg, sampledf.vg$Canola.Lines)# Homogenity of
dispersion test
permutest(beta)## was  significant - so we can  reject the null hypothessis that
canola genotypes have the same disperssion.
#we did not proceed for correlating mean bray distance with genetic distance since
we have not observed significant variation in bray distance.

## Flowering stage
can.Flowering.2016 = subset_samples(phyloseq.object.corrected.line.names.2016,
Week=="4"|Week=="5"|Week=="6"|Week=="7") ## subset to weeks 4,5,6,7:Flowering stage

can.Flowering.2016.edgernorm = normalise_data(can.Flowering.2016, norm.method =
"edgernorm")## normalize the data using edgeR normalization

#PERMANOVA ON edgeR method normalized data using adonis: using phyloseq #

set.seed(100)
can_bray.2016.fl <-phyloseq::distance(can.Flowering.2016.edgernorm,
                                       method = "bray")# calculate bray curtis
distance matrix
sampledf.fl = data.frame(sample_data(can.Flowering.2016.edgernorm))# make a data
frame from the sample_data of the phyloseq object
adonis(can_bray.2016.fl~Canola.Lines, data = sampledf.fl) # perform permanova test
using adonis function in Vegan ## was  significant- rejecting that genotypes have
the same centroid
beta = betadisper(can_bray.2016.fl, sampledf.fl$Canola.Lines)# Homogenity of
dispersion test
permutest(beta)## was not significant - so we can not reject the null hypothessis
that canola genotypes have the same disperssion.
```

```
#### calculate mean bray-curtis distance between each pair of genotypes : then
correlate it with genetic distance

#Calculate mean distance between genotypes

can_bray_mean.fl = meandist(can_bray.2016.fl, sampledf.fl$Canola.Lines)##
calculates mean distance between canola lines
head(can_bray_mean.fl) ## this is a dist object, so first change it into matrix
library(reshape) # you need this to export the distance measures in data.frame
format
can_bray_mean_matrix.fl = as.matrix(can_bray_mean.fl) # change it into matrix
m3 <-
melt(can_bray_mean_matrix.fl)[melt(upper.tri(can_bray_mean_matrix.fl))$value,] #
use melt, upper.tri from reshape
names(m3) <- c("c1", "c2", "distance") # give name to the columns
m3 # check the out put

write.table(m3,'D:/out_puts/phyloseq/with_green_genes_taxa/microbiomeseq_analyssi/c
an_bray_mean_line.2016.flowering.csv', sep = ",") # write the output as csv file
# plot, correlate plant genetic distance + bray distane
library(ggpubr)

# creat a data frame manually with mean bray dis and genetic dist
#bray curtis- genetic distance correlation based on flowering data, all line pairs
bray_genetic_distance_all_lines_Flowering <-
read_excel("D:/out_puts/phyloseq/with_green_genes_taxa/microbiomeseq_analyssi/bray_
genetic_distance_all_lines_Flowering.xlsx")
p12= ggscatter(bray_genetic_distance_all_lines_Flowering, x = "Genetic.Distance", y
= "Mean.Bray.Curtis",
               add = "reg.line", conf.int = TRUE,
               cor.coef = TRUE, cor.method = "pearson",
               xlab="", ylab = "")## scater plot with trend line and correlation
coefficient with p value
p12= p12 +theme(axis.title.x = element_text(size = 14), axis.title.y =
element_text(size = 14))
p12=p12+theme(axis.text.x = element_text(size=12), axis.text.y=element_text(size =
12))
p12 ## positive and significant correlation

## Maturity

can.maturity.2016 = subset_samples(phyloseq.object.corrected.line.names.2016,
Week=="8"|Week=="9"|Week=="10") ## subset to weeks 8. 9,10 - maturity stage

can.maturity.2016.edgernorm = normalise_data(can.maturity.2016, norm.method =
"edgernorm")## normalize the data using edgeR normalization

#PERMANOVA ON edgeR method normalized data using adonis: using phyloseq #

set.seed(100)
```

```
can_bray.2016.ma <-phyloseq::distance(can.maturity.2016.edgernorm,
                                      method = "bray")# calculate bray curtis
distance matrix
sampledf.ma = data.frame(sample_data(can.maturity.2016.edgernorm))# make a data
frame from the sample_data of the phyloseq object
adonis(can_bray.2016.ma~Canola.Lines, data = sampledf.ma) # perform permanova test
using adonis function in Vegan ## was not significant- Acepting that genotypes have
the same centroid
beta = betadisper(can_bray.2016.ma, sampledf.ma$Canola.Lines)# Homogenity of
dispersion test
permutest(beta)## was not significant - so we can not reject the null hypothessis
that canola genotypes have the same disperssion.

#we did not proceed for correlating mean bray distance with genetic distance since
we have not observed significant variation in bray distance


#### Weeks 1-7(vegetative to flowering)
can.Veg.Flow.2016 = subset_samples(phyloseq.object.corrected.line.names.2016,
Week=="1"| Week=="2"| Week=="3"|Week=="4"|Week=="5"|
                                     Week=="6"|Week=="7") ## subset to weeks1,2,3
4,5,6,7:vegetative+Flowering stage


can.Veg.Flow.2016.edgernorm = normalise_data(can.Veg.Flow.2016, norm.method =
"edgernorm")## normalize the data using edgeR normalization

set.seed(100)
can_bray.2016.vg.fl <-phyloseq::distance(can.Veg.Flow.2016.edgernorm,
                                      method = "bray")# calculate bray curtis
distance matrix
sampledf.vg.fl = data.frame(sample_data(can.Veg.Flow.2016.edgernorm))# make a data
frame from the sample_data of the phyloseq object
adonis(can_bray.2016.vg.fl~Canola.Lines, data = sampledf.vg.fl) # perform permanova
test using adonis function in Vegan ## was  significant- rejecting that genotypes
have the same centroid
beta = betadisper(can_bray.2016.vg.fl, sampledf.vg.fl$Canola.Lines)# Homogenity of
dispersion test
permutest(beta)## was not significant - so we can not reject the null hypothessis
that canola genotypes have the same disperssion.


#### calculate mean bray-curtis distance between each pair of genotypes : then
correlate it with genetic distance

#Calculate mean distance between genotypes
can_bray_mean.vg.fl = meandist(can_bray.2016.vg.fl, sampledf.vg.fl$Canola.Lines)##
calculates mean distance between canola lines
head(can_bray_mean.vg.fl) ## this is a dist object, so first change it into matrix
library(reshape) # you need this to export the distance measures in data.frame
```

```
format
can_bray_mean_matrix.vg.fl = as.matrix(can_bray_mean.vg.fl) # change it into matrix
m4 <-
melt(can_bray_mean_matrix.vg.fl)[melt(upper.tri(can_bray_mean_matrix.vg.fl))$value,
] # use melt, upper.tri from reshape
names(m4) <- c("c1", "c2", "distance") # give name to the columns
m4 # check the out put

write.table(m4,'D:/out_puts/phyloseq/with_green_genes_taxa/microbiomeseq_analyssi/c
an_bray_mean_line.2016.vegetative.flowering.csv', sep = ",") # write the output as
csv file
# plot and correlate genetic distance between reference line and genotypes with
bray distane between reference line and genotypes
library(ggpubr)

# creat a data frame manually with mean bray dis and genetic dist
##bray curtis- genetic distance correlation based on vegetative-flowering data, all
line pairs
bray_genetic_distance_all_lines_vegetative_flowering <-
read_excel("D:/out_puts/phyloseq/with_green_genes_taxa/microbiomeseq_analyssi/bray_
genetic_distance_all_lines_vegetative_flowering.xlsx")
p13= ggscatter(bray_genetic_distance_all_lines_vegetative_flowering, x =
"Genetic.Distance", y = "Mean.Bray.Curtis",
               add = "reg.line", conf.int = TRUE,
               cor.coef = TRUE, cor.method = "pearson",
               xlab="Genetic distance", ylab = "Mean Bray-Curtis")## scater plot
with trend line and correlation coefficient with p value
p13= p13 +theme(axis.title.x = element_text(size = 14), axis.title.y =
element_text(size = 14))
p13=p13+theme(axis.text.x = element_text(size=12), axis.text.y=element_text(size =
12))
p13 ## positive and significant correlation

####and weeks 4-10 (flowering to maturity)

can.Flow.matu.2016 = subset_samples(phyloseq.object.corrected.line.names.2016,
Week=="4"|Week=="5"|

Week=="6"|Week=="7"|Week=="8"|Week=="9"|Week=="10") ## subset to weeks
4,5,6,7,8,9,10:Fowering+maturity stage

can.Flow.matu.2016.edgernorm = normalise_data(can.Flow.matu.2016, norm.method =
"edgernorm")## normalize the data using edgeR normalization

set.seed(100)
can_bray.2016.fl.ma <-phyloseq::distance(can.Flow.matu.2016.edgernorm,
                                         method = "bray")# calculate bray curtis
distance matrix
sampledf.fl.ma = data.frame(sample_data(can.Flow.matu.2016.edgernorm))# make a data
frame from the sample_data of the phyloseq object
```

```
adonis(can_bray.2016.fl.ma~Canola.Lines, data = sampledf.fl.ma) # perform permanova
test using adonis function in Vegan ## was  significant- rejecting that genotypes
have the same centroid
beta = betadisper(can_bray.2016.fl.ma, sampledf.fl.ma$Canola.Lines)# Homogenity of
dispersion test
permutest(beta)## was not significant - so we can not reject the null hypothessis
that canola genotypes have the same disperssion.



#### calculate mean bray-curtis distance between each pair of genotypes : then
correlate it with genetic distance

#Calculate mean distance between genotypes
can_bray_mean.fl.ma = meandist(can_bray.2016.fl.ma, sampledf.fl.ma$Canola.Lines)##
calculates mean distance between canola lines
head(can_bray_mean.fl.ma) ## this is a dist object, so first change it into matrix
library(reshape) # you need this to export the distance measures in data.frame
format
can_bray_mean_matrix.fl.ma = as.matrix(can_bray_mean.fl.ma) # change it into matrix
m5 <-
melt(can_bray_mean_matrix.fl.ma)[melt(upper.tri(can_bray_mean_matrix.fl.ma))$value,
] # use melt, upper.tri from reshape
names(m5) <- c("c1", "c2", "distance") # give name to the columns
m5 # check the out put

write.table(m5,'D:/out_puts/phyloseq/with_green_genes_taxa/microbiomeseq_analyssi/c
an_bray_mean_line.2016.flowering.maturity.csv', sep = ",") # write the output as
csv file

# creat a data frame manually with mean bray dis and genetic dist
##bray curtis- genetic distance correlation based on flowering-maturity data, all
line pairs
bray_genetic_distance_all_lines_Flowering_maturity <-
read_excel("D:/out_puts/phyloseq/with_green_genes_taxa/microbiomeseq_analyssi/bray_
genetic_distance_all_lines_Flowering_maturity.xlsx")
p14= ggscatter(bray_genetic_distance_all_lines_Flowering_maturity, x =
"Genetic.Distance", y = "Mean.Bray.Curtis",
               add = "reg.line", conf.int = TRUE,
               cor.coef = TRUE, cor.method = "pearson",
               xlab="Genetic distance", ylab = "")## scater plot with trend line
and correlation coefficient with p value
p14= p14 +theme(axis.title.x = element_text(size = 14), axis.title.y =
element_text(size = 14))
p14=p14+theme(axis.text.x = element_text(size=12), axis.text.y=element_text(size =
12))
p14 ## positive and significant correlation
```
