# Supplementary material for: Core and Differentially Abundant Bacterial Taxa in the Rhizosphere of Field Grown Brassica napus Genotypes: Implications for Canola Breeding
Source: Front Microbiol. 2020 Jan 15;10:3007. doi: 10.3389/fmicb.2019.03007 (PMC6974584; doi:10.3389/fmicb.2019.03007)
Supplement: Supplementary file 4 [file Data_Sheet_4.docx]

Supplementary Materials

**Summary**

**Supplementary tables**

Table S1. Total number of sequence reads per sample, *B. napus* line and week.

Table S2. List of the thirty-two bacterial phyla identified in the *B. napus* rhizosphere in 2016 and 2017 experiment at Saskatoon. The number of amplicon sequence variants (ASVs) observed for each phylum (frequency), sum of prevalence of each ASV within a phylum divided by corresponding frequency (mean prevalence), and total abundance of each phylum are presented. Unique phyla among the two years are in bold.

Table S3. Total abundance of the three major phyla per sample. Column top phylum indicates the phylum most abundant in respective samples.

Table S4. Summary of the number of reads for the three major bacterial phyla (Acidobacteria, Actinobacteria, and Proteobacteria) in each of the sixteen *B. napus* genotypes in 2016 experiment. N (number of samples for each line), average, minimum, maximum, and total reads are presented.

Table S5. *B. napus* core bacterial taxa identified at three sites and two years with their corresponding prevalence and relative abundances (relative to total number of reads observed at each site or location).

Table S6. List of differentially abundant bacterial taxa in each *B. napus* line compared with the reference (NAM-0) and their associated log fold change (logFC), log count per million (logCPM), false discovery rate (FDR) and p-value.

**Supplementary figures**

Figure S1. Rhizosphere vs bulk soil. Rhizosphere soil is defined as the soil that remained attached to the root after shaking of the loosely held bulk soil.

Figure S2. Prevalence and abundance distribution of amplicon sequence variants (ASVs) within each phylum. Each point represents an individual ASV. Total abundance is in log (10) scale and the prevalence in the Y axis is prevalence of each ASVs divided by number of samples.

Figure S3. Dendrogram plot based on single nucleotide polymorphisms across the genomes of sixteen *B. napus* genotypes determined using an Illumina chip.

Figure S4. The top twenty differentially abundant bacterial genera based on log fold change (logFC) in fifteen *B. napus* genotypes compared with the reference (NAM-0).

Table S1. Total number of sequence reads per sample, *B. napus* line and week.

**Please find a separately attached file.**

Table S2. List of the thirty-two bacterial phyla identified in the *B. napus* rhizosphere in 2016 and 2017 experiment at Saskatoon. The number of amplicon sequence variants (ASVs) observed for each phylum (frequency), sum of prevalence of each ASV within a phylum divided by corresponding frequency (mean prevalence), and total abundance of each phylum are presented. Unique phyla among the two years are in bold.

|  | 2016 Experiment | | | 2017 experiment | | |
| --- | --- | --- | --- | --- | --- | --- |
| Phylum | Frequency | Mean prevalence (%) | Total abundance | Frequency | Mean prevalence (%) | Total abundance |
| Acidobacteria | 1674 | 10.95 | 302574116 | 789 | 2.68 | 3615078 |
| Actinobacteria | 1469 | 9.81 | 255740786 | 922 | 4.63 | 25411969 |
| Armatimonadetes | 37 | 1.76 | 434147 | 42 | 1.40 | 26622 |
| Bacteroidetes | 1190 | 4 | 56370358 | 1374 | 3.64 | 39823950 |
| BRC1 | 58 | 2.74 | 1308494 | 15 | 1.00 | 5600 |
| Chlamydiae | 2 | 1 | 213 | 2 | 1.50 | 697 |
| Chlorobi | 27 | 1.56 | 259023 | 24 | 1.13 | 11211 |
| Chloroflexi | 1158 | 8.18 | 179006987 | 478 | 2.25 | 1285903 |
| Cyanobacteria | 23 | 2.39 | 514495 | 9 | 1.00 | 3443 |
| Elusimicrobia | 21 | 1 | 21040 | 44 | 1.05 | 17622 |
| **FBP** | 1 | 1 | 418 | na | na | na |
| Fibrobacteres | 11 | 1.27 | 23326 | 9 | 1.00 | 2909 |
| Firmicutes | 121 | 5.65 | 7833364 | 115 | 4.95 | 2455646 |
| **GAL15** | 3 | 1.33 | 9269 | na | na | na |
| Gemmatimonadetes | 464 | 4.92 | 22796090 | 269 | 2.56 | 1035027 |
| Kazan-3B-28 | 3 | 1 | 724 | 1 | 1.00 | 112 |
| MVP-21 | 5 | 1 | 4162 | 1 | 1.00 | 471 |
| Nitrospirae | 79 | 9.08 | 7097647 | 47 | 1.89 | 61309 |
| NKB19 | 13 | 1.38 | 32353 | 1 | 1.00 | 78 |
| OD1 | 16 | 1.75 | 149248 | 17 | 1.12 | 5426 |
| Planctomycetes | 287 | 4.42 | 11125654 | 320 | 2.06 | 1216201 |
| Proteobacteria | 4655 | 6.15 | 679739753 | 3186 | 4.01 | 221735149 |
| **SR1** | 1 | 1 | 545 | na | na | na |
| Tenericutes | 1 | 1 | 743 | 2 | 1.00 | 344 |
| Thermi | 7 | 1.57 | 16822 | 3 | 1.00 | 644 |
| TM6 | 5 | 1 | 17131 | 9 | 1.22 | 2886 |
| TM7 | 285 | 2.44 | 6061954 | 237 | 1.75 | 458557 |
| unclassified | 333 | 1.04 | 446149 | 69 | 1.06 | 21157 |
| Verrucomicrobia | 545 | 4.3 | 39155387 | 447 | 2.00 | 1242451 |
| WPS-2 | 3 | 1.67 | 7454 | 9 | 1.22 | 4005 |
| WS2 | 16 | 2.31 | 68743 | 2 | 1.00 | 416 |
| WS3 | 54 | 2.56 | 617164 | 28 | 1.71 | 30105 |
| **AD3** | Na | Na | na | 3 | 2.67 | 3493 |
| **BHI80-139** | Na | Na | na | 1 | 1.00 | 351 |
| **GN02** | Na | Na | na | 2 | 1.00 | 383 |

Table S3. Total abundance of the three major phyla per sample. Column top phylum indicates the phylum most abundant in respective samples.

**Please find a separately attached file.**

Table S4. Summary of the number of reads for the three major bacterial phyla (Acidobacteria, Actinobacteria, and Proteobacteria) in each of the sixteen *B. napus* genotypes in 2016 experiment. N (number of samples for each line), average, minimum, maximum, and total reads are presented.

**Please find a separately attached file.**

Table S5. *B. napus* core bacterial taxa identified at three sites and two years with their corresponding prevalence and relative abundances (relative to total number of reads observed at each site or location).

**Please find a separately attached file.**

Table S6. List of differentially abundant bacterial taxa in each *B. napus* line (named separate sheets) compared with the reference (NAM-0) and their associated log fold change (logFC), log count per million (logCPM), false discovery rate (FDR) and p-value.

**Please find a separately attached file.**


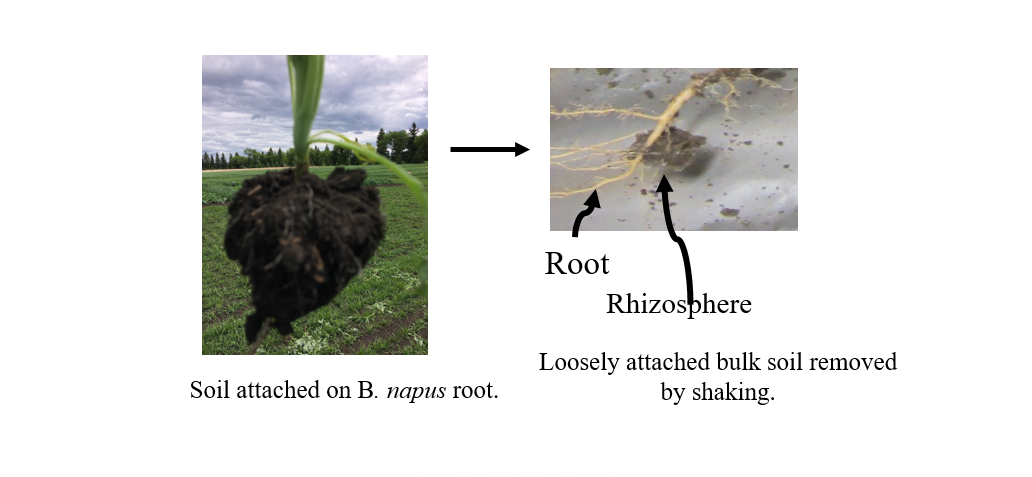


Figure S1. Rhizosphere vs bulk soil. Rhizosphere soil is defined as the soil that remained attached to the root after shaking of the loosely held bulk soil.


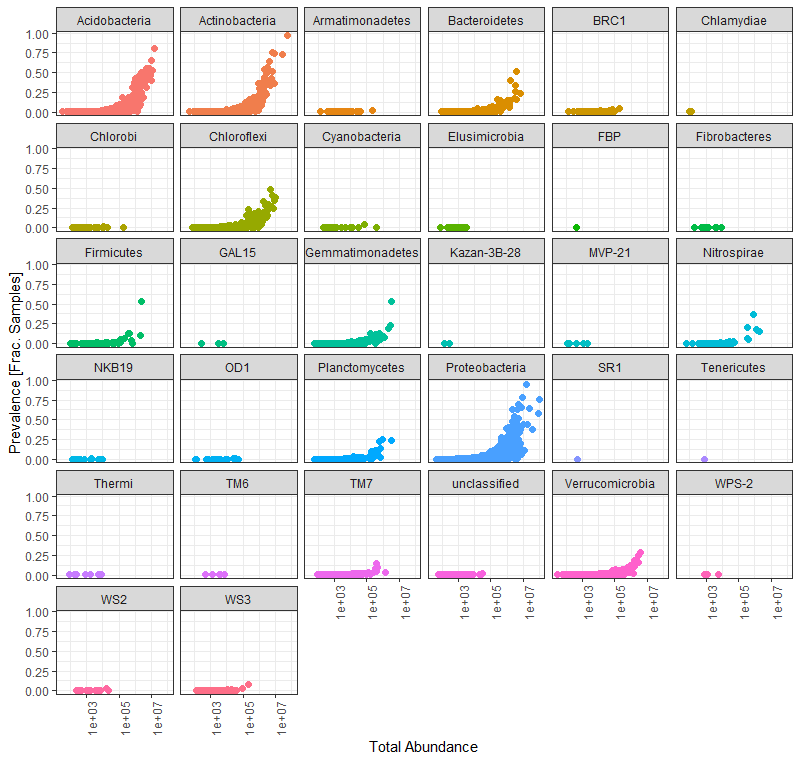


Figure S2. Prevalence and abundance distribution of amplicon sequence variants (ASVs) within each phylum. Each point represents an individual ASV. Total abundance is in log (10) scale and the prevalence in the Y axis is prevalence of each ASVs divided by number of samples.


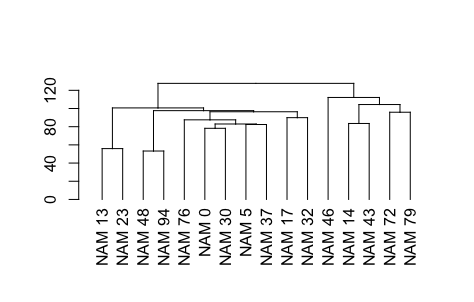


Figure S3. Dendrogram plot based on single nucleotide polymorphisms across the genomes of sixteen *B. napus* genotypes determined using an Illumina chip.


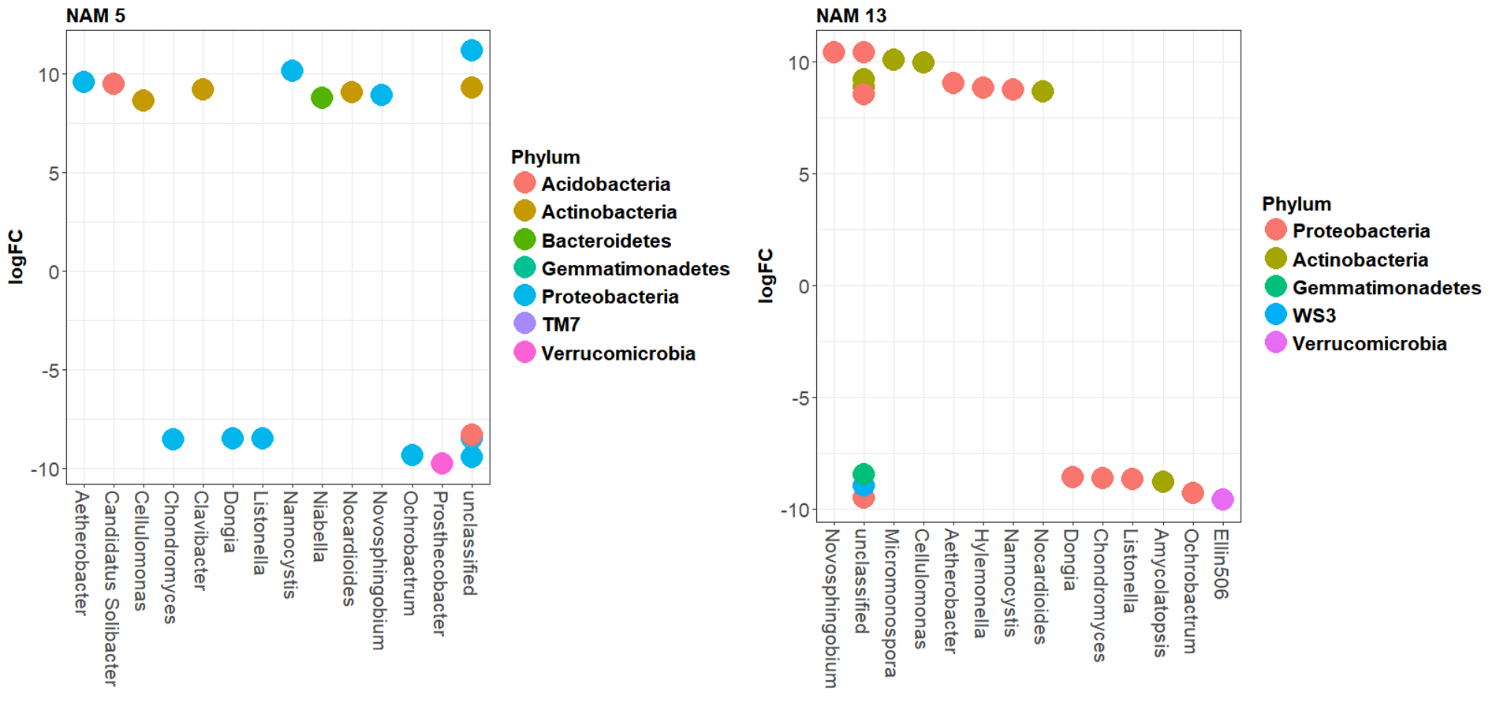


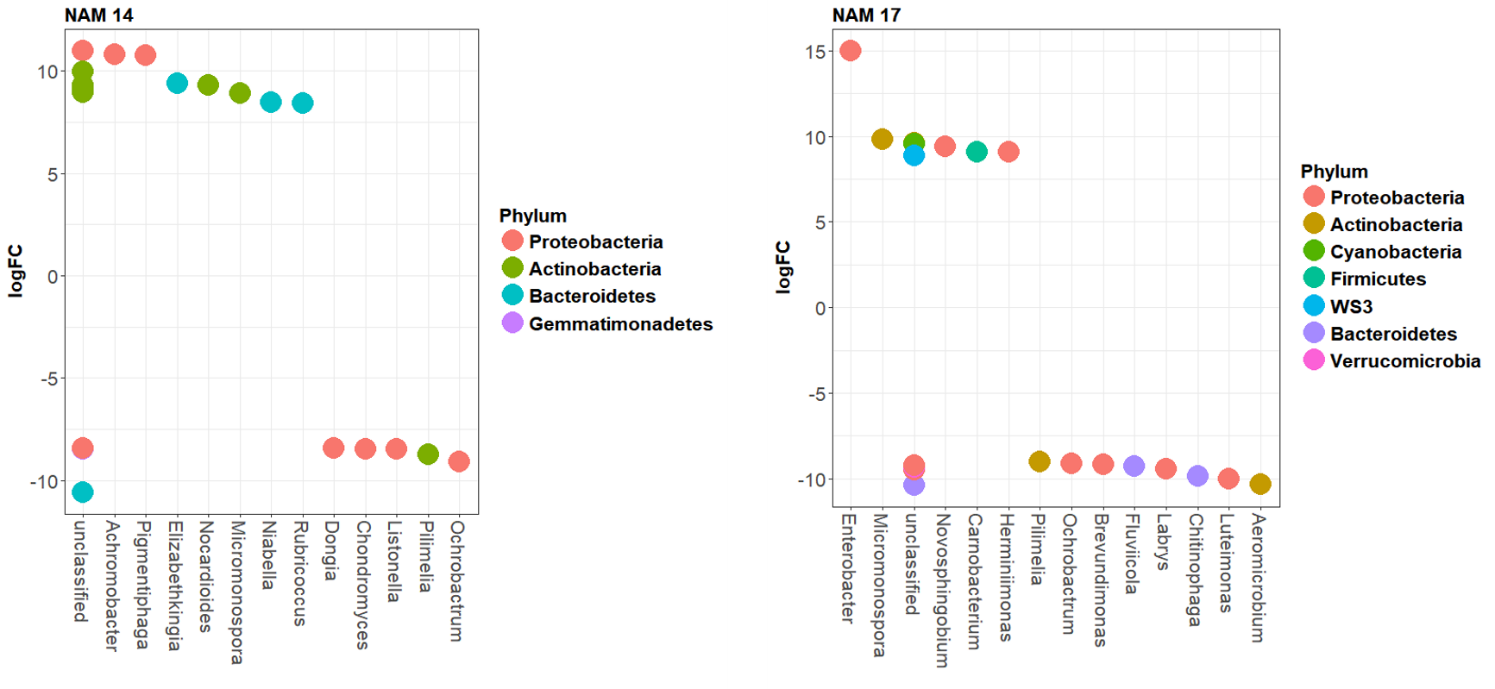


Figure S4. The top twenty differentially abundant bacterial genera based on log fold change (logFC) in fifteen *B. napus* genotypes compared with the reference (NAM-0).


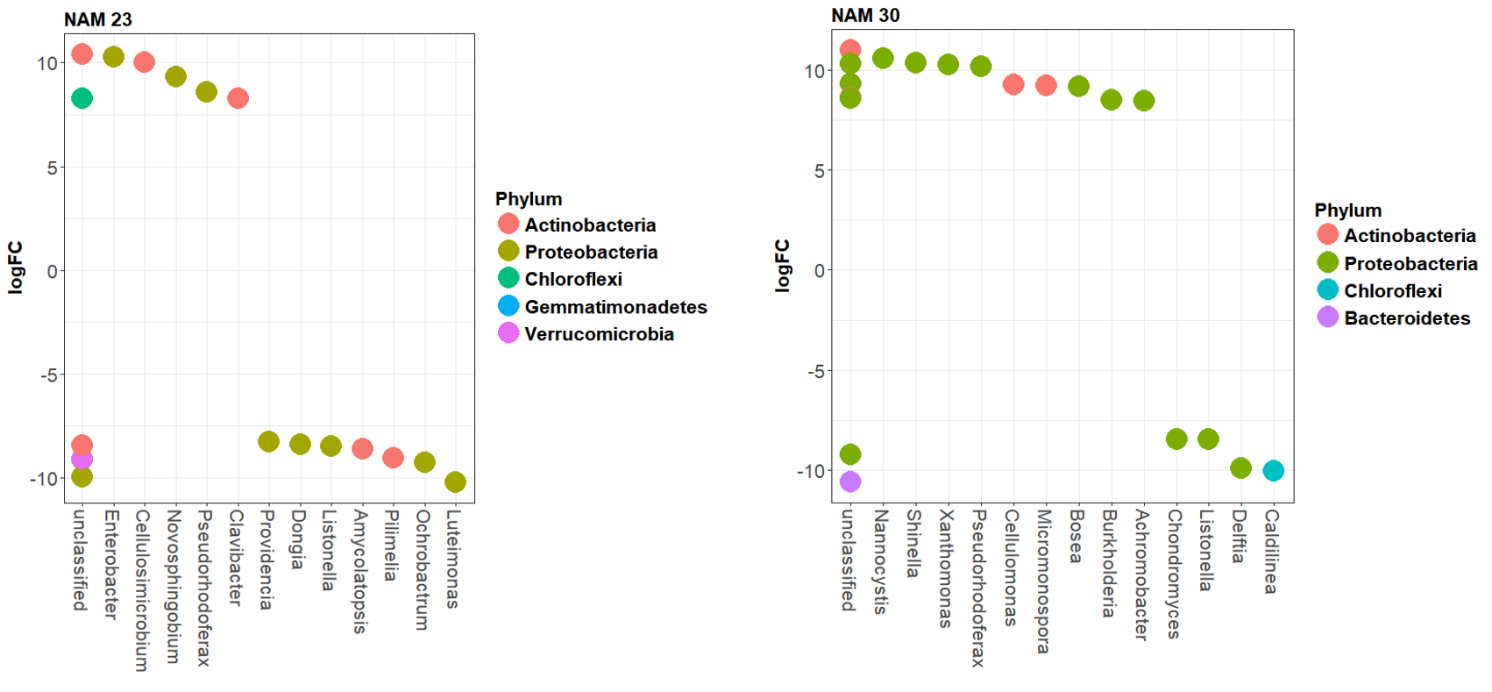


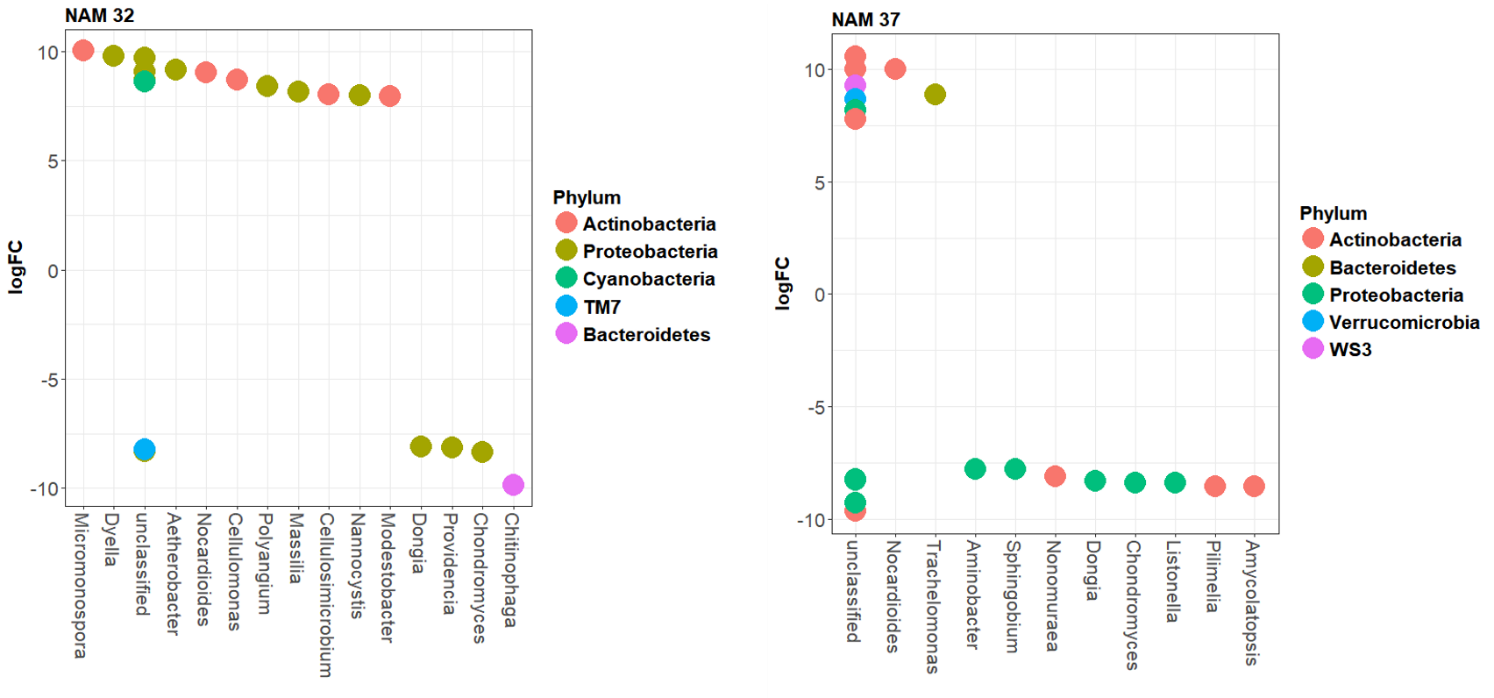


Figure S4. The top twenty differentially abundant bacterial genera based on log fold change (logFC) in fifteen *B. napus* genotypes compared with the reference (NAM-0).


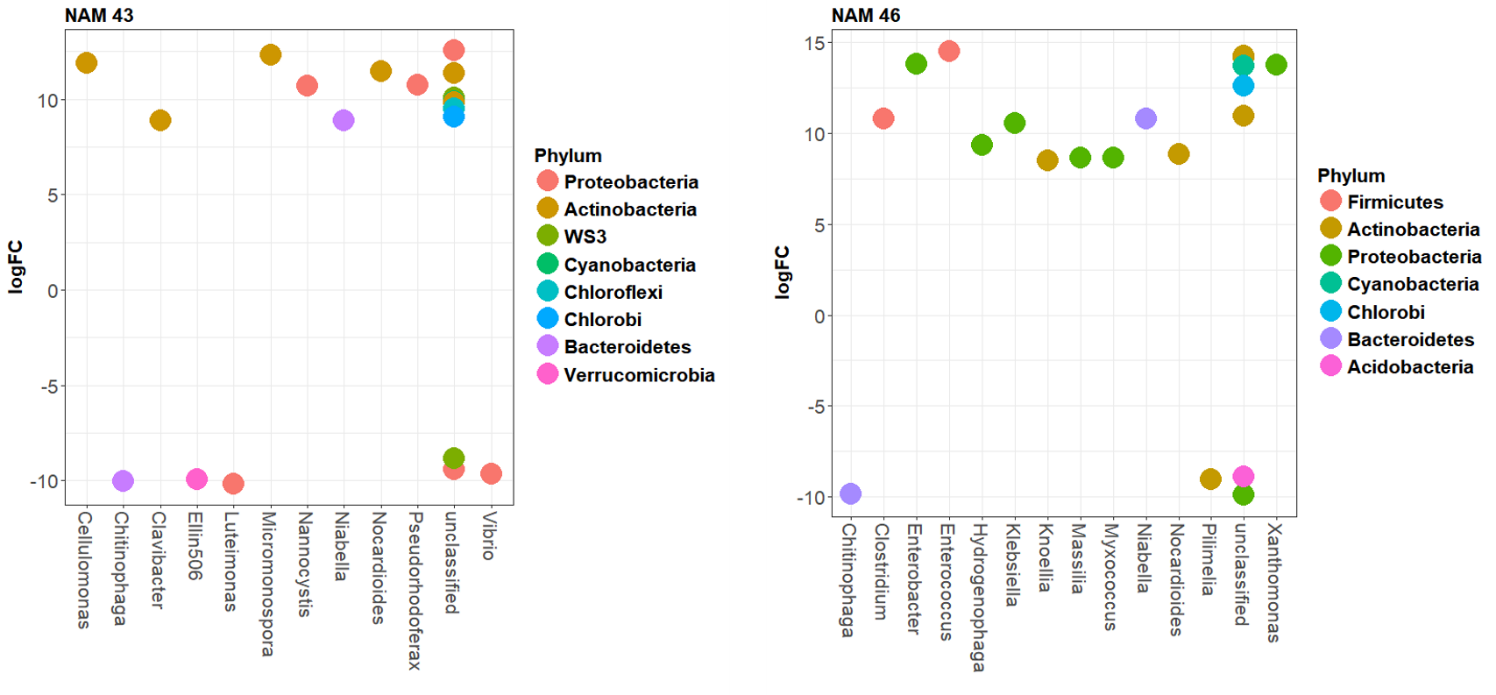


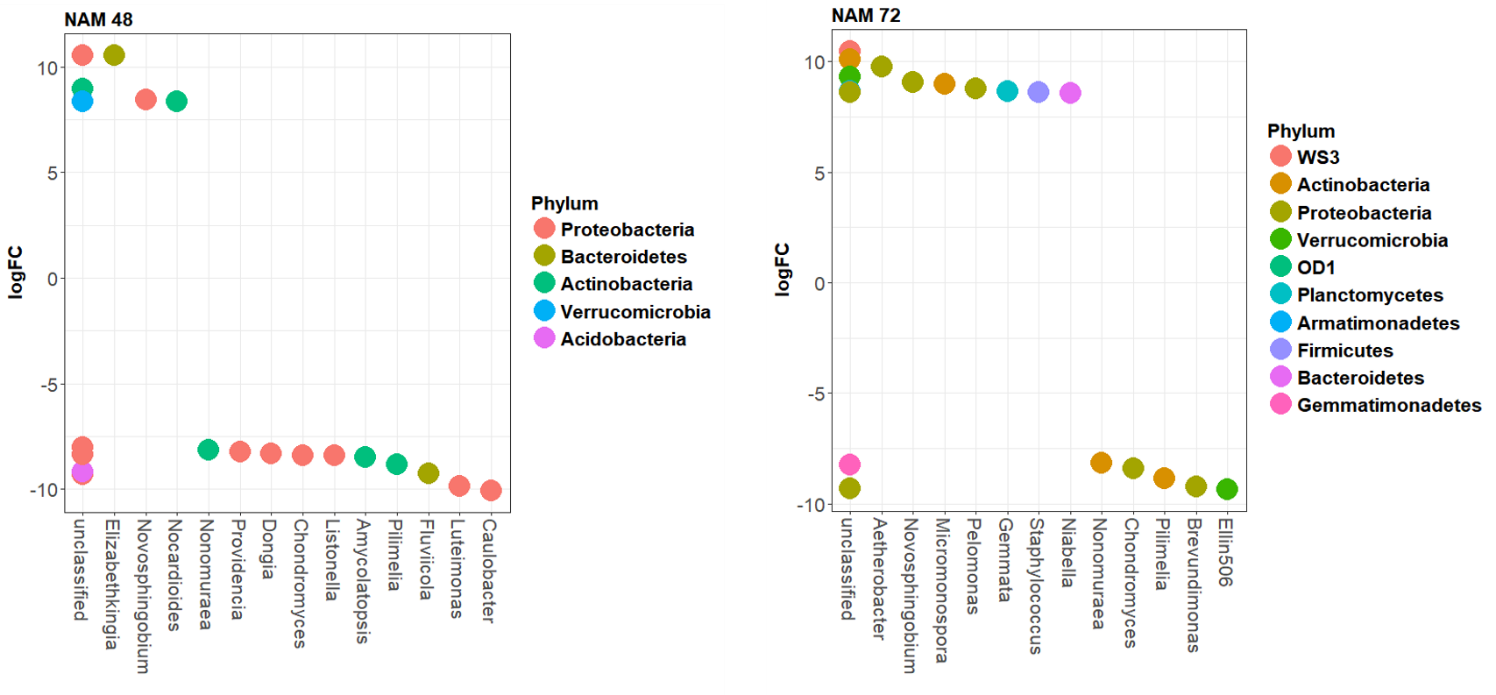


Figure S4. The top twenty differentially abundant bacterial genera based on log fold change (logFC) in fifteen *B. napus* genotypes compared with the reference (NAM-0).


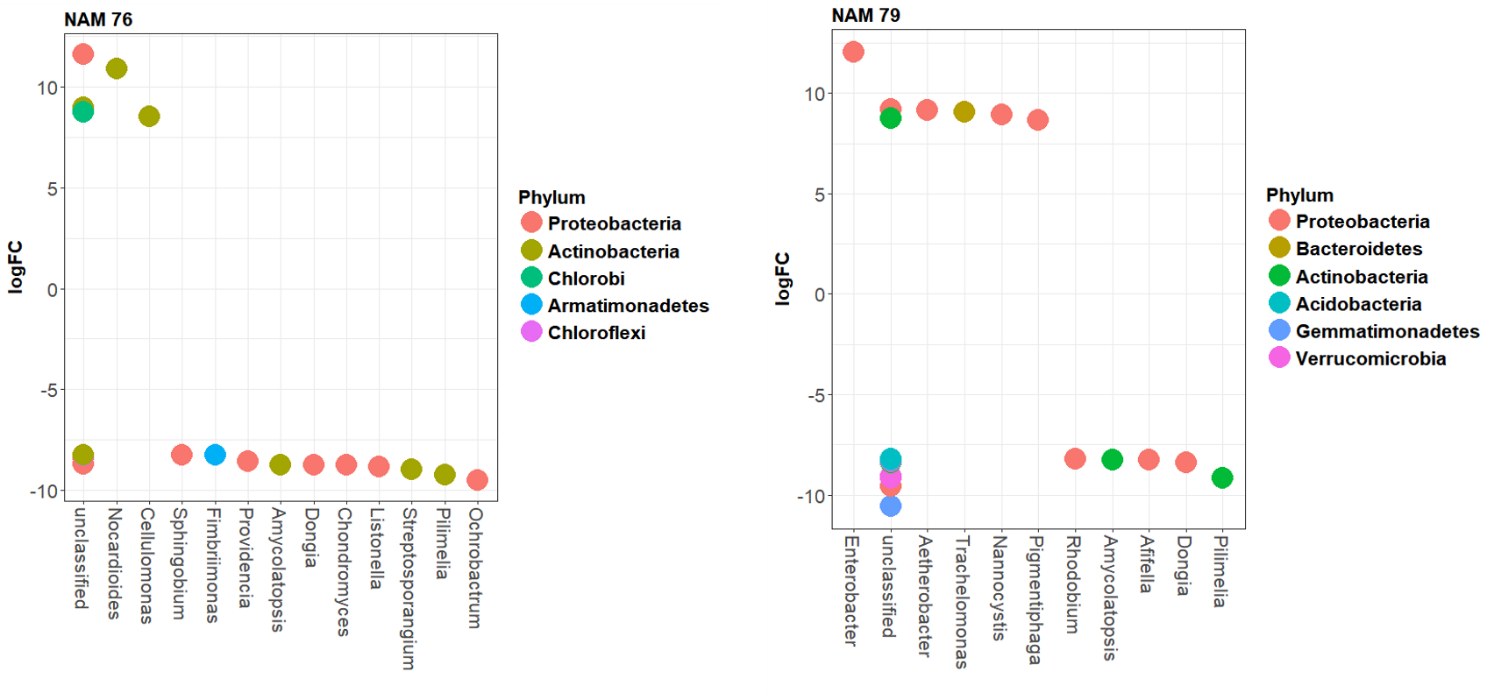


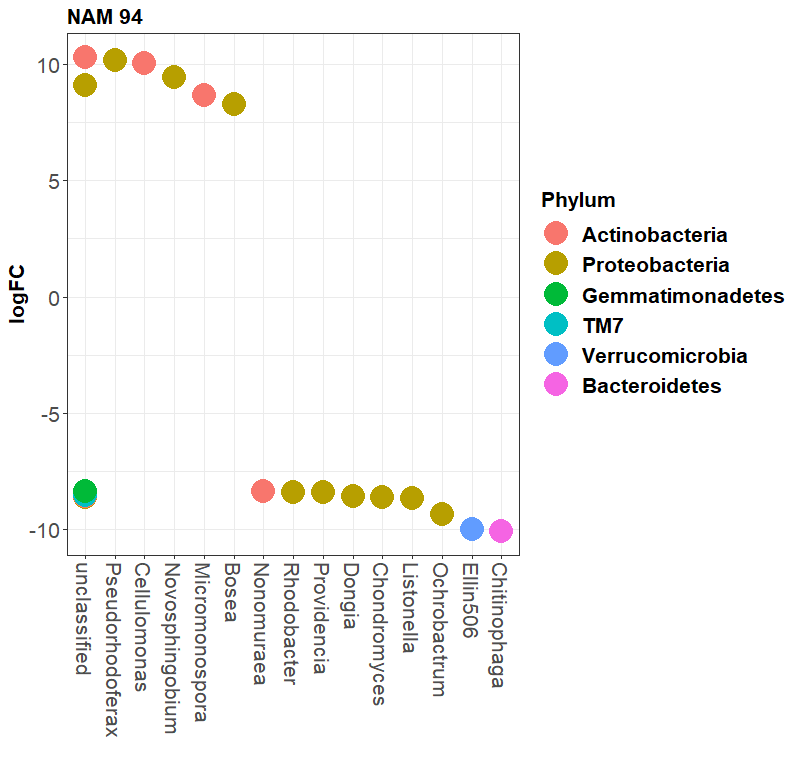


Figure S4. The top twenty differentially abundant bacterial genera based on log fold change (logFC) in fifteen *B. napus* genotypes compared with the reference (NAM-0).
